# Potential Inhibitors for Novel Coronavirus Protease Identified by Virtual Screening of 606 Million Compounds

**DOI:** 10.3390/ijms21103626

**Published:** 2020-05-21

**Authors:** André Fischer, Manuel Sellner, Santhosh Neranjan, Martin Smieško, Markus A. Lill

**Affiliations:** Computational Pharmacy, Department of Pharmaceutical Sciences, University of Basel, 4056 Basel, Switzerland; and.fischer@unibas.ch (A.F.); manuel.sellner@unibas.ch (M.S.); santhosh.neranjan@stud.unibas.ch (S.N.)

**Keywords:** coronavirus, SARS-2-CoV, virtual screening, computational chemistry

## Abstract

The rapid outbreak of the novel severe acute respiratory syndrome coronavirus 2 (SARS-CoV-2) in China followed by its spread around the world poses a serious global concern for public health. To this date, no specific drugs or vaccines are available to treat SARS-CoV-2 despite its close relation to the SARS-CoV virus that caused a similar epidemic in 2003. Thus, there remains an urgent need for the identification and development of specific antiviral therapeutics against SARS-CoV-2. To conquer viral infections, the inhibition of proteases essential for proteolytic processing of viral polyproteins is a conventional therapeutic strategy. In order to find novel inhibitors, we computationally screened a compound library of over 606 million compounds for binding at the recently solved crystal structure of the main protease (M^pro^) of SARS-CoV-2. A screening of such a vast chemical space for SARS-CoV-2 M^pro^ inhibitors has not been reported before. After shape screening, two docking protocols were applied followed by the determination of molecular descriptors relevant for pharmacokinetics to narrow down the number of initial hits. Next, molecular dynamics simulations were conducted to validate the stability of docked binding modes and comprehensively quantify ligand binding energies. After evaluation of potential off-target binding, we report a list of 12 purchasable compounds, with binding affinity to the target protease that is predicted to be more favorable than that of the cocrystallized peptidomimetic compound. In order to quickly advise ongoing therapeutic intervention for patients, we evaluated approved antiviral drugs and other protease inhibitors to provide a list of nine compounds for drug repurposing. Furthermore, we identified the natural compounds (−)-taxifolin and rhamnetin as potential inhibitors of M^pro^. Rhamnetin is already commercially available in pharmacies.

## 1. Introduction

In late 2019, a novel coronavirus, termed severe acute respiratory syndrome coronavirus 2 (SARS-CoV-2) was determined as a cause for several cases of respiratory disease in China. Even though most infected patients only suffer from mild symptoms such as fever and cough associated with a good prognosis, the disease can progress into fatal cases of pneumonia and acute respiratory failure, especially in older people with comorbidities [1,2]. The virus rapidly spread from China to over 141 countries worldwide and, up to this date, infected almost two million people claiming more than 100,000 fatalities [3] (as of 14 April 2020), already exceeding the 2003 SARS-CoV epidemic [4]. Most probably, the virus originated from a zoonotic transmission between animals such as bats and humans but progressed to transmit from human to human through common droplet infection [2,5,6]. The World Health Organisation declared the SARS-CoV-2 outbreak a Public Health Emergency of International Concern [7,8] and, based on the 2003 epidemic of SARS-CoV, it will lead to the loss of many lives exerting enormous social impact and economic loss [9]. Clinical treatment of the disease is mainly symptomatic and based on repurposing of already marketed antiviral drugs such as ritonavir and antibiotics to treat secondary infections [1]. Thus, there remains an urgent need for the development of specific antiviral therapeutics and vaccines against SARS-CoV-2 [4,10,11,12].

The inhibition of viral proteases necessary for proteolytic processing of polyproteins has been a successful strategy in the pharmacological treatment of human immunodeficiency virus (HIV) and hepatitis C virus, respectively [13,14], proving the potential of protease inhibitors for the treatment of viral infections. Similarly, the main protease (M^pro^) of SARS-CoV-2 is thought to be essential for viral replication and, therefore, is regarded as promising target for antiviral pharmacotherapy [10,15]. The crystal structure of the SARS-CoV-2 main protease was recently solved [16] which enables the rational design of specific inhibitory compounds. The close relationship of SARS-CoV-2 to SARS-CoV is reflected by the high sequence identity of 96.1% among their M^pro^ [10,17]. In this regard, it was suggested that compounds developed against SARS-CoV might be effective to treat SARS-CoV-2 [12] and can therefore be considered as template structures for M^pro^ inhibitors. However, these compounds remained in the preclinical or early clinical stage and did not lead to approved therapeutics [18]. Furthermore, their effectiveness for the novel virus might suffer due to differences in individual amino acids [19] as we discuss in the beginning of the next section. Accordingly, the development of specific inhibitors for SARS-CoV-2 main protease remains an urgent necessity in the scientific community [10,11,12] which is reflected by the multiple projects focusing on this protein. For example, three studies investigating the repurposing of marketed drugs proposed several candidates for SARS-CoV-2 treatment [10,15,20,21]. In another recent study, a deep learning approach based on a fully connected neural network trained on the PDBBind database [22] combined with a homology model of the protease was applied to screen a library of approximately one million compounds including already approved drugs, tripeptides, and natural products [12]. However, the disadvantage of applying therapeutics originally designed for a different target is the risk of undesired pharmacological effects and adverse reactions [23]. Even though vaccine development can be assisted by computational methods [24], this study is focused on the design of a small-molecule enzyme inhibitor.

In this study, we screened a large library of over 606 million compounds with the aim to discover novel inhibitors for the SARS-CoV-2 main protease. We designed a protocol consisting of a combination of intensively validated methods that were successfully applied in drug discovery programs either as standalone tools or in combination [25,26,27,28]. In a first step, we performed a shape-based screening with known binders for the SARS-CoV main protease and relevant substructures as template molecules. After the initial shape screening, two different docking protocols were applied followed by the assessment with pharmacokinetic filters to narrow down the number of potential binders. Clustering based on molecular fingerprints was applied to ensure structural diversity of the compounds that were, in the next step, subjected to molecular dynamics (MD) simulations. Based on the obtained trajectories, the binding free energy of the ligands was quantified using Molecular Mechanics/Generalized Born Surface Area (MM/GBSA) post-processing. In the last step, we assessed potential toxic effects of the compounds due to the interaction with 16 known off-targets to make a final selection of 12 compounds. In addition, we report the highest scored natural compounds and a list of marketed drugs that could be repurposed for SARS-CoV-2 treatment. Such a comprehensive exploration of chemical space intending to discover SARS-CoV-2 M^pro^ inhibitors was, to the best of our knowledge, not previously reported.

## 2. Results and Discussion

### 2.1. Comparison of Proteases between SARS-CoV-2 and SARS-CoV

**Figure 1:**
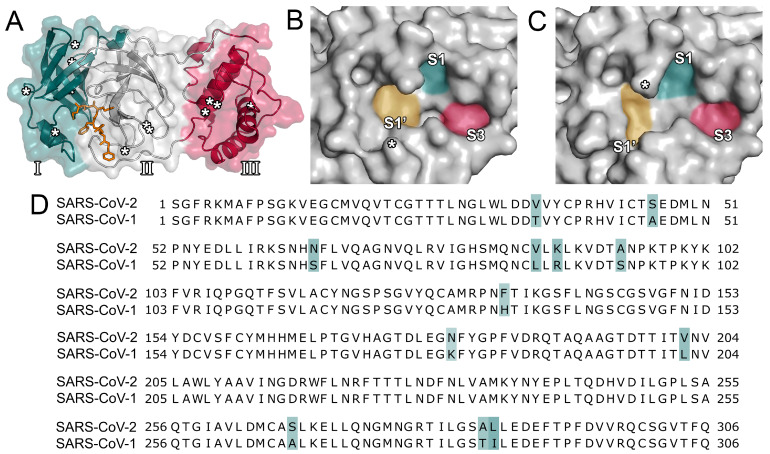
Structural overview and sequence alignment. (**A**) The three domains (domain I in blue, domain II in grey, domain III in red, as indicated by roman numerals) of the main protease of severe acute respiratory syndrome coronavirus 2 (SARS-CoV-2) are shown. Amino acid changes between SARS-CoV and SARS-CoV-2 are indicated by asterisks. The cocrystallized ligand (PDB ID 6LU7) is presented in orange. (**B**) Surface topology of the binding pocket of the SARS-CoV-2 main protease (PDB ID 6LU7). The location of Ser46 is indicated by an asterisk. (**C**) Surface topology of the binding pocket of the SARS-CoV main protease (PDB ID 2A5I). The location of Asn142 is indicated by an asterisk. (**D**) Sequence alignment of the proteases of SARS-CoV and SARS-CoV-2. Mismatches are marked in blue.

The SARS-CoV-2 M^pro^ protease consists of three domains (Figure 1A) [18] and processes polyproteins using histidine (His41) and cysteine (Cys145) as catalytic residues. Its active site is located between domains I and II. As SARS-CoV-2 is closely related to SARS-CoV, their proteases display a high degree of sequence similarity (96.1%; Figure 1D) [10,17]. In the vicinity of the active site, only a single amino acid (Ser46) is different among the two proteases. However, the surface topology of the active site among the two proteins presents distinct differences, especially in the vicinity of the loop centered around Asn142 (Figure 1B,C). Additionally, the size and depth of the S1’ pocket shows notable differences and, at the center of the S1, S1’, and S3, the SARS-CoV-2 protease presents a more distinct subcavity as opposed to the SARS-CoV enzyme. Consequently, inhibitors of the SARS-CoV protease might display altered binding affinities for the SARS-CoV-2 protease. Similarities to other viral proteases such as the one of HIV or Middle-East Respiratory Syndrome coronavirus (MERS-CoV) are comparatively low [29]. Although the main protease presumably is the most promising therapeutic target to attenuate viral replication, the inhibition of other functional proteins such as the papain-like protease or the interaction between the viral spike protein and its entry receptor to human cells were considered as well [8,29].

### 2.2. Virtual Screening Procedures

**Figure 2:**
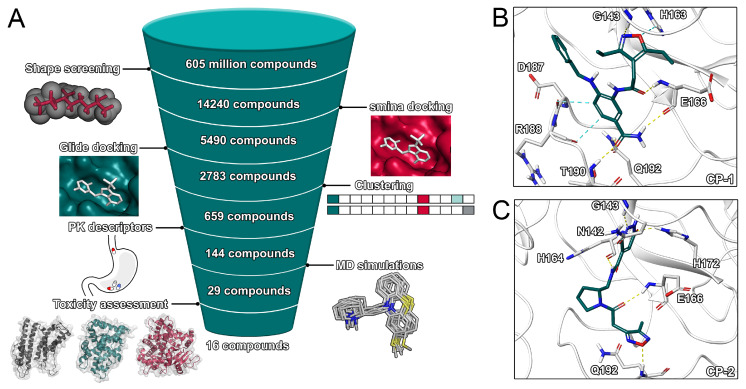
Main virtual screening workflow and binding poses of top two ligands. (**A**) Virtual screening workflow. (**B**) Binding pose of CP-1. (**C**) Binding pose of CP-2.

Virtual Screening is a widely used technique at the early stage of drug discovery that allows to identify potentially bioactive compounds at a high throughput [30]. In several prior projects, coalescing different virtual screening methods lead to the discovery of potent inhibitors [25,26,27,31]. Due to its speed and cost-effectiveness, it is a promising approach to identify potential drug candidates against the globally expanding SARS-CoV-2 virus, especially when time is of essence. The inhibition of proteases as treatment against viral infections has been proven for HIV and HCV, which renders the SARS-CoV-2 main protease an attractive drug target, in particular since its crystal structure has been recently determined. For our virtual screening efforts (Figure 2 and Figure S1, Supporting Information), we extracted a total of 606 million compounds from the ZINC database. First, all compounds in three-dimensional form were screened with respect to their shape similarity [25] against a pre-selected set of known and co-crystalized SARS-CoV and SARS-CoV-2 inhibitors. Such a coarse GPU-accelerated protocol allows for the rapid screening of large databases based on known binders as template. Previous virtual screening efforts were limited to significantly smaller compound libraries [10,12,15,17,20].

In our main screening workflow (Figure 2), we refined the high number of initial hits (1,122,542 compounds) to 14,240 compounds by selecting the best hits regarding shape overlap. The remaining compounds were docked into the active site of five representative structures of the protease using the experimentally validated [28] smina docking protocol. While the majority of compounds was scored with a value below −5.0 kcal/mol, 5490 potential hits were selected based on a score below the defined threshold of −7.0 kcal/mol. While most previous studies regarding SARS-CoV-2 were limited to a homology model of the protease [10,12,15,17], our study relied on a recently solved crystal structure for our docking procedures. The potential for induced fit effects was determined to be comparably small (Figure S2).

In the attempt to increase confidence in ligand ranking based on docking scores, we used Glide SP as second docking protocol to evaluate the interaction of the remaining 5490 compounds with the binding site of the protease. The protocol was previously applied to discover experimentally validated inhibitors of the SARS-CoV papain-like protease [32]. Except for two compounds, a valid binding pose was detected in all cases with most compounds scoring below −5.0 kcal/mol. In order to validate the docking protocols, we performed cross-docking for 63 cocrystallized ligands to our structural ensemble (Table S1) and determined root-mean-square deviation (RMSD) values between experimental and predicted poses. The RMSD values ranged from 1.2 to 9.6 Å with peaks around 2 and 5 Å (Figures S4 and S5). The performance of the smina docking protocol regarding pose prediction was slightly superior. The set of compounds with a Glide score below −6.5 kcal/mol was comprised of a high number of congeneric ligands including many structurally similar natural compounds. Therefore, in order to increase the structural diversity of the compound set used for subsequent calculations, we computed extended connectivity fingerprints and clustered the compounds according to the Tanimoto coefficient. For each cluster, the two compounds with the best Glide score were selected and evaluated regarding their pharmacokinetic properties. The calculation of pharmacokinetic descriptors is discussed in the Supporting Information. Compounds were not selected if they violated either the Lipinski or Veber criteria. This selection process resulted in 144 compounds used for final MD simulations and free-energy calculations. Based on the resulting MD trajectories of the ligand–protein complexes, the binding free energies of the ligands were estimated using the MM/GBSA protocol. This protocol was recently applied to predict the interaction energy between nelfinavir and the SARS-CoV-2 main protease [10] as well as to other screening projects [26]. Compounds with a predicted binding free energy better than the cocrystallized ligand were finally selected. Those 29 compounds were characterised by their potential toxicity. The VirtualToxLab evaluates the toxic potential of a small molecule on the basis of individual binding affinities to 16 validated off-targets including nuclear receptors, metabolic enzymes, and the human Ether-à-go-go-Related Gene potassium channel (hERG). Such an assessment at an early stage of drug discovery might mitigate the attrition rate of drugs due to toxicity and safety which represent a large share of preclinical and clinical failures of drug development programs [33]. The final 29 compounds included 13 compounds with a toxic potential above 0.5 that were discarded from the final set. We also investigated the two natural compounds with the lowest predicted binding free energy, which were (−)-taxifolin and rhamnetin.

In addition to the ligand library extracted from ZINC, we also repeated the screening process on a library of 1.4 million compounds from ZINC database with a MW above 500 g/mol (Figure S1). This screening process resulted in 38 compounds subjected to MD simulations and 19 compounds used for toxicity profiling by the VirtualToxLab (Table S3). The molecular descriptors relevant for pharmacokinetic evaluation were not considered in this second screening run. In this context, it must be mentioned that for reliable individual molecule interaction data on physical-chemical basis, numerical tools that do not assume any a priori model should be used [34].

### 2.3. Final Compound Selection

**Figure 3:**
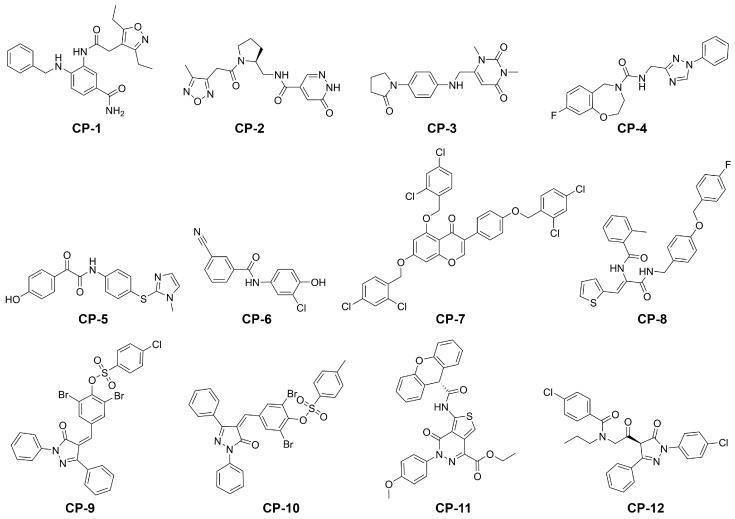
Structures of our final selection of compounds.

**Figure 4:**
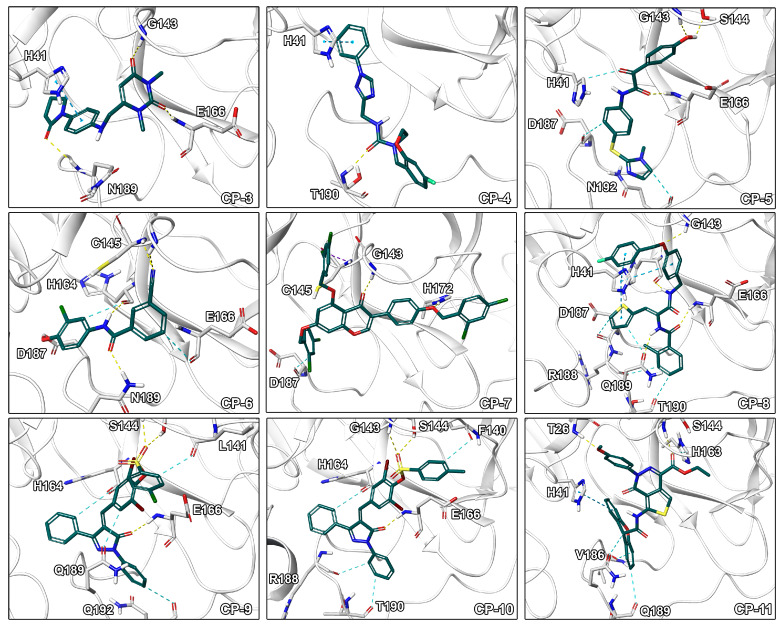
Binding modes of CP-3 to CP-11. Ligand-protein interactions are shown as dashed lines with hydrogen bonds color coded in yellow, aromatic and π-π interactions in blue, halogen bonds in purple, and salt bridges in pink.

In summary, both screening processes identified 28 compounds with a lower predicted free energy of binding. For 16 of those potential hits the potential of absorption after oral administration was predicted to be higher than than of the cocrystallized ligand N3 (Tables S2 and S3). For each of the two screening processes, we selected the six best potential hits according to their predicted binding free energy (Table 1 and Figure 3). The reported set of ligands should be regarded as early lead compounds since no experimentally supported optimization was conducted. The experimental evaluation of binding affinity and kinetics could be conducted, for example, by using techniques such as surface plasmon resonance (SPR) or quartzcrystal microbalance (QCM) coupled to a robust statistical model addressing the inherent complexity of biomolecular interactions [34]. While some compounds only showed a slight decrease with respect to the predicted binding energy compared to the cocrystallized ligand N3, several others presented a substantial improvement of up to 55%. Our proposed antivirals interacted with the target with at least one hydrogen bond with an average of over three (Figure 1A,B and Figure 4, Figure S6 and Table S4). Furthermore, the proposed hits from the main screen (MW below 500 g/mol) displayed excellent pharmacokinetic descriptors with logD values ranging from −1.9 to 3.3, MW below 406.5 g/mol, PSA below 129.8 Å^2^ and hydrogen bond acceptors as well as donors in the allowed range described by Lipinski [35]. In contrast to other proposed inhibitors for SARS-CoV-2 [18], the availability of compounds is denoted as either for sale, in stock, or on demand in the ZINC database (Tables S2 and S3). Compounds CP-1, CP-3, CP-4, CP-5, and (−)-taxifolin displayed comparatively low predicted toxicity values and therefore represent excellent candidates for further in vitro investigations. The binding profile of these compounds is depicted in Figure S7. Natural compounds such as flavonoids were previously tested as inhibitors of the main protease of SARS-CoV and their effectiveness was demonstrated [18,36]. The Persian walnut (Juglans regia) and japanese cypress (Chamaecyparis obtusa) among others are known to produce glycosides of (−)-taxofolin offering a natural resource for its extraction. Glycosylated flavonoids regularly show an increased bioavailability compared to the aglycon [37]. In the organism, these glycosides are cleaved by β-glucosidase in the gastrointestinal tract or liver [38,39]. Since the aglycon already exhibits acceptable pharmacokinetics in rabbits [40], glycoyslated (−)-taxifolin will likely display improved bioavailability as well. Remarkably, the aglycon engaged in six direct hydrogen bonds with the M^pro^ which represents the highest count observed in our selection of compounds, which offers a naturally occurring alternative to the proposed inhibitors CP-1 to CP-12. However, the isomer (+)-taxifolin, which is more common in plants, was predicted to have less favourable binding free energies (Table S5). The second highest scoring natural compound in our main screening, rhamnetin, occurs in Moringa oleifera among other plants [41,42]. Rhamnetin is suggested to have improved pharmacokinetics compared to the structurally similar natural compound quercetin for which a half-life of over three hours was determined in humans [43,44]. Furthermore, preparations containing rhamnetin are readily available from pharmacies and other companies allowing direct and fast access to the potential antiviral.

**Figure 5:**
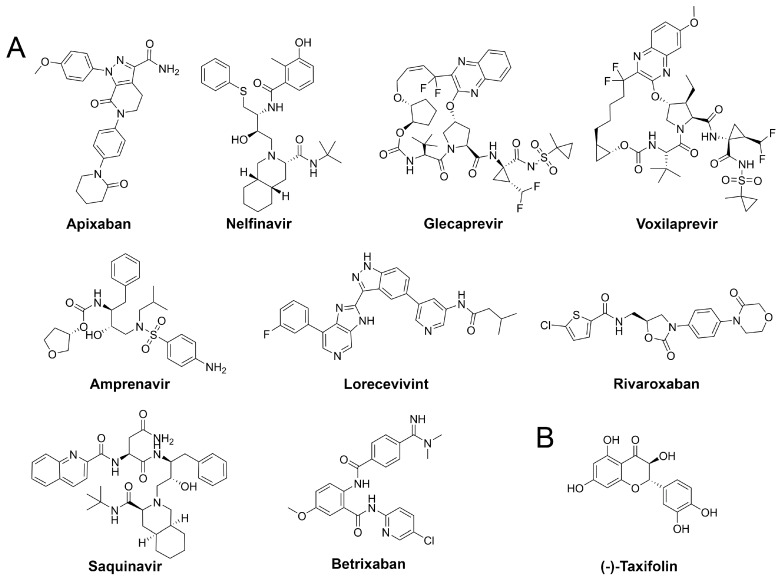
Structures of drug repurposing hits and the highest scored natural compound.

**Figure 6:**
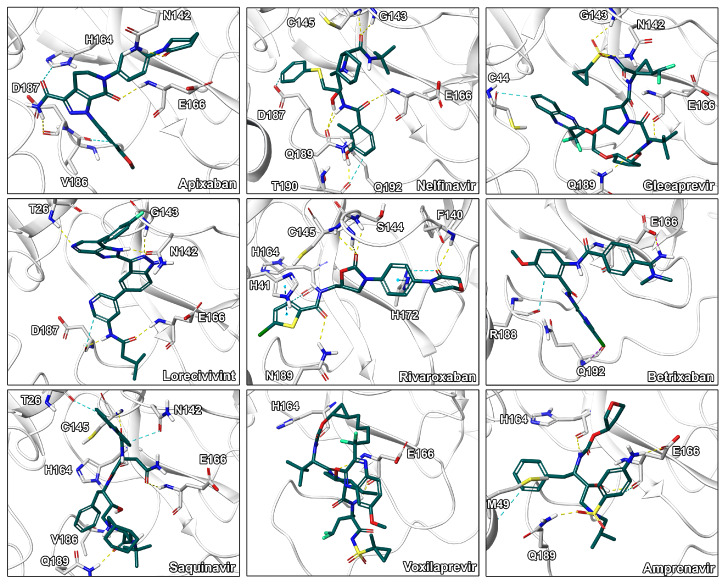
Binding modes of the drug repurposing hits. Ligand-protein interactions are shown as dashed lines with hydrogen bonds are shown in yellow, aromatic and π-π interactions in blue, halogen bonds in purple, and salt bridges in pink.

Our assessment of commercially available drugs revealed multiple candidates with improved predicted free energy of binding compared to the cocrystallized inhibitor N3 (Table 2 and Table S6, Figure 5). Except for one, all of these compounds were approved for human pharmacotherapy. The top-ranked compounds based on predicted free energy of binding are the factor Xa inhibitor apixaban [45], and the two known antivirals nelfinavir and glecaprevir [46,47]. Visual inspection of the binding pose of apixaban revealed a high complementarity to the protein and most heteroatoms engaged in ligand–protein interactions along with a deeply buried hydrogen bond formed by its terminal amide bond (Figure 6). Since anticoagulants are already administered to patients suffering from coronavirus infections due to associated coagulopathy [48], the selection of an appropriate factor Xa inhibitor could further improve the benefit of such therapeutic interventions. Nelfinavir was previously suggested as a potential SARS-CoV-2 inhibitor in computational and cellular studies [10,49]. For example, a screening of 1903 small-molecule drugs predicted nelfinavir as the most promising compound using both MM/GBSA and solvated interaction energy (SIE) scoring [10] which confirms our high ranking of this ligand. The binding pose of nelfinavir presented seven hydrogen bonds with reasonable complementarity to the protease binding pocket. The only non-approved compound originating from the DrugBank similarity search was lorecivivint, which is currently investigated for osteoarthritis treatment [50]. Other anticoagulants such as rivaroxaban and betrixaban presented comparable binding free energies in our analysis.

Ligand efficiency, a measure derived from scaling affinities by molecular size, is a widely used design parameter in drug discovery. Even though the concept is criticized due to its dependence on the used concentration unit used to report affinity, we determined the ligand efficiency of our lead compounds [52] (Table 1 and Table 2). All top-ranked compounds obtained from our virtual screening process show an improved ligand efficieny compared to N3 (Table 1). The ligand efficiency of CP-12 (−3.7 kcal/mol) was predicted to be even more than two-fold higher that that of the cocrystallized inhibitor N3 (−1.6 kcal/mol). In addition, multiple screening hits displayed improved predicted ligand efficiency compared to the most efficient commercially available factor Xa inhibitor apixaban. Compounds with high ligand efficiency coupled to excellent pharmacokinetic descriptors include CP-2, CP-3, and CP-6.

Visual inspection of binding modes is regularly conducted by computational medicinal chemists to select appropriate candidates for synthesis or experimental testing [30,53]. During this evaluation, we identified several poses with high complementarity to the binding pocket including deeply buried hydrophobic residues, extensive ligand–protein interactions as well as a high degree of aromatic hydrogen bonds (Figure 4). Especially, interactions to the catalytic residue His41 would be of high value since an amino acid exchange at this position potentially interferes with the mechanism of the enzyme as it was shown for SARS-1-CoV by site-directed mutagenesis experiments [54]. Indeed, CP-8 offered a good fit to the pocket including a deeply buried thiophene moiety, multiple π-π stacking interactions with His41, as well as only few heteroatoms lacking polar contacts. This indicates that CP-8 may be a good candidate, identified in the high MW screening process, for further experimental evaluation. Similar interaction patterns were observed for CP-3 originating from the main screening process. Further, CP-2 presented several hydrogen bonds to backbone atoms of the protein indicating high stability of the interactions against structural fluctuations.

## 3. Materials and Methods

### 3.1. System Preparation and Ensemble Generation

The protein structure of the SARS-CoV-2 main protease was retrieved from the Protein Data Bank (PDB ID 6LU7) and processed using the Protein Preparation Wizard that comes with the Maestro Small-Molecule Drug Discovery Suite (v2019-4) [55]. The Protein Preparation Wizard adds hydrogen atoms, predicts protonation states at a pH of 7.4, and optimizes the hydrogen bonding network. Sequence alignment to the protease of SARS-CoV was performed using the Ugene suite of tools combined with the ClustalW algorithm [56,57]. Next, the system was subjected to restrained minimization with an RMSD convergence threshold of 0.3 Å using the OPLS_2005 force field. To obtain an ensemble of structures for following procedures, an MD simulation with a duration of 20 ns was performed using the Desmond (v2019-1) simulation engine [58]. To solvate the orthorhombic periodic boundary systems extending 10 Å from the complex in every dimension, we selected the TIP3P water model. First, the default relaxation protocol of Desmond was executed, followed by the production phase at 310 K in an NPT ensemble. For all production simulations in this study, we applied the Martyna–Tobias–Klein barostat, with a relaxation time of 2.0 ps and the Nose–Hoover thermostat, with a relaxation time of 1.0 ps. We used the u-series algorithm to treat long-range interactions with a cutoff of 9 Å for short-range interactions [59]. The M-SHAKE algorithm is used to constrain bonds to hydrogen atoms. The timestep of the RESPA integrator was set to 2.0 fs and snapshots with atomic coordinates were saved at an interval of 10 ps. Using the resulting simulation trajectory, we used the trj_cluster.py script provided in Maestro, which relies on an affinity propagation algorithm, to determine five representative structures of the protease.

### 3.2. Pharmacokinetic Descriptors

Chemical descriptors for the Lipinski and Veber rules [35], as well as the aqueous solubility (logS), were determined using the cxcalc module of the Marvin (v20.4.0) suite [60]. Those properties have significant influence on the pharmacokinetic properties of drug candidates. Instead of the commonly used logP value, we decided to use the logD value which allows to predict the partitioning of ligands with respect to variable ionization states [61]. Properties dependent on the ionization state of a compound were computed at a physiological pH value of 7.4. The descriptors were computed for all 6428 ligands that were supplied to the Glide docking protocol in both screening runs.

### 3.3. Docking and Shape Screening

**Figure 7:**
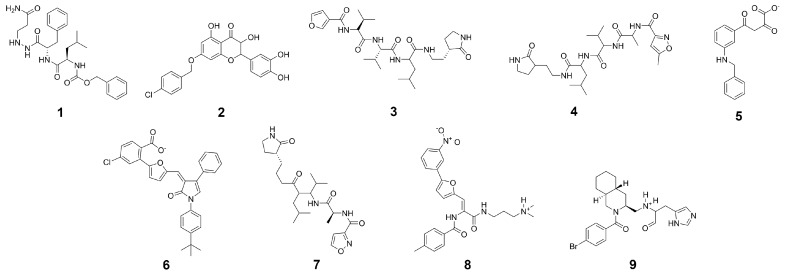
Input structures for shape screening derived from crystal structures and the PubChem database.

In the first step of our virtual screening process, the GPU-accelerated shape screening protocol of Maestro was employed on the drug-like portion of the ZINC [62] library. Additionally, due to the comparatively large size of the cocrystallized peptidomimetic inhibitor, compounds with a molecular weight above 500 g/mol were used as query molecules. As templates, a substructure of the cocrystallized ligand of SARS-CoV-2 protease (compound **4**), a cocrystallized ligand (compound **9**) and three substructures of the equivalent SARS-CoV protease inhibitors (compounds **1**, **3**, and **7**), as well as four known SARS-CoV inhibitors with binding affinity below 10 μM (compounds **2**, **5**, **6**, and **8**) based on the annotated K_i_ or IC_50_ values derived from the PubChem database [63] were selected (Figure 7). From these experimentally verified inhibitors [64,65,66,67] four out of a set of 14 compounds (Table S7) were selected as templates based on their structural diversity and due to the structural similarity of the above-mentioned main proteases of SARS-CoV and SARS-CoV-2. In detail, molecular extended connectivity fingerprints were used for characterizing each compound, similarity between compounds was determined based on the Tanimoto similarity coefficient, and subsequent clustering in the Discovery Informatics panel within Maestro was performed to select the representative template structures. The template structures were put through the LigPrep routine in Maestro where protonation states were determined at a pH of 7.4. Shape similarity between all query and template molecules was determined. Potential hit compounds were selected, if the best shape overlap with any template molecules exceeded a threshold of 45%. The best 12,000 hits regarding shape similarity, as well as the top 600 compounds with respect to each template were selected for the subsequent screening steps. In addition, the top 200 compounds were included in the case of a shape similarity for multiple template structures. Screening of 1,402,468 compounds with a molecular weight above 500 g/mol was performed separately according to the above described workflow. Violations of Lipinski and Veber rules were not taken into consideration for this library (Figure S1). Only compounds with a predicted binding free energy below were considered as potential hits. Toxicity profiling was only performed on this subset of molecules.

The flexibility of the binding site residues of M^pro^ was analyzed on nine cocrystal structures from the Protein Data Bank (PDB) [68] and 58 structures published by Diamond [69]. The structures were superimposed with the Protein Structure Alignment panel in Maestro [55]. Next, we used the smina [70] docking protocol, a fork of AutoDock Vina (v1.1.2) [71], on the above-mentioned compounds to quantify their interaction with each of the representatives structures from the generated ensemble. The ligand PDBQT files for docking were generated using the prepare_ligand4.py script that it part of the AutoDockTools suite (v1.5.4) [72]. Docking was performed at an exhaustiveness of 16, the search space was defined using the automatic method with smina, and a random seed of 42 was selected. Potential hits with a binding free energy below −7.0 kcal/mol were then subjected to the default Glide standard precision (SP) docking protocol in Maestro [73]. The interaction score with all five ensemble structures was quantified. Compounds with a minimum free binding energy below −6.5 kcal/mol were then subjected to clustering based on the Tanimoto comparison of per-atom molecular fingerprints. For each of the 306 resulting clusters, the best two compounds (one compound in the case of a single structure per cluster) according to the Glide score were selected for further assessment. The commercial availability of the compounds was retrieved from the ZINC15 website. Compounds were selected for the next step according to the following criteria: (i.) availability not orphaned, (ii.) no violations of Lipinski and Veber criteria, (iii.) average ensemble score below −6.6 kcal/mol in smina docking, (iv.) average ensemble score below −6.0 kcal/mol in Glide SP docking, (v.) sum of best docking scores in either protocol below −14.5 kcal/mol, (vi.) best smina score below −7.2, (vii.) best Glide score below −7.0, and (viii.) logS value of −6 or higher. The same docking and ranking procedure was performed with the cocrystallized ligand N3, as well as 63 crystal structures to assess cross-docking performance (Table S1). MD simulations were performed on this representative set of potential hit compounds.

### 3.4. MD Simulations and Post-Processing

In order to more precisely quantify the interaction energies of the previously filtered set of ligands, we conducted MD simulations of each compound. As input for the simulations, we chose the ligand–protein complex with the highest Glide SP docking score among the ensemble of structures. Simulations were run for 25 ns with atomic coordinates recorded at an interval of 25 ps, while the remaining settings were the same as those described above in context of ensemble generation.

The binding free energy was quantified using the MM/GBSA methodology with the thermal_mmgbsa.py routine as part of the Maestro software suite. The last 100 frames of the trajectory with a step size of two were chosen for the MM/GBSA calculation. MM/GBSA calculations were performed for apo and holo states of the enzyme; only enthalpic components and implicit (de)solvation were included in those calculations. The resulting free energies were averaged for the selected frames of the trajectories. The binding free energy was finally determined by the difference of averaged free energies between holo and apo state. The ligand efficiency was determined by dividing the averaged MM/GBSA scores through the number of heavy atoms of the respective compound.

### 3.5. Drug Repurposing

From a search on PubChem [63], several protease inhibitors including commercially available HIV or hepatitis C antivirals [46,47,74,75] were chosen. Protease inhibitors targeting metalloproteases were not considered. Furthermore, we conducted a similarity search of all DrugBank molecules [51] using FP4 fingerprints against known cocrystallized competitive antivirals that bind to SARS-CoV and SARS-CoV-2 (Figure S8). To compute the FP4 fingerprints and compare them according to the Tanimoto coefficient, we used OpenBabel (v3.0.0) [76]. The most similar compound for each of the 19 input structures was selected for further processing. All drug compounds were again subjected to both docking protocols followed by MD and MM/GBSA post-processing. In total, MD simulations with a length of 5.38 μs were performed in this study.

### 3.6. Computational Off-Target Profiling and Selection of Final Set of Compounds

Potential toxic effects of the compounds that were subjected to MD simulations were assessed using the VirtualToxLab [23,77] that evaluates the binding affinity of a probe compound to 16 known off-targets. The target structures include androgen receptor (AR), aryl hydrocarbon receptor (AhR), cytochrome P450 1A2 (CYP1A2), CYP2C9, CYP2D6, CYP3A4, estrogen receptor α (ERα), ERβ, glucocorticoid receptor (GR), mineralocorticoid receptor (MR), liver X receptor (LXR), hERG channel, peroxisome proliferator-activated receptor γ (PPARγ), progesterone receptor (PR), thyroid receptor α (TRα), and TRβ. The routine quantifies the binding affinities to each target and, based on them, estimates a numerical value of toxicity termed toxic potential ranging from 0.0 to 1.0 and values above 0.6 indicate high toxicity. The results of VirtualToxLab were previously shown to correlate well with experimental data [78].

The final set of potential hit compounds was selected based on their toxic potential and average free binding energy from MM/GBSA. Compounds with an MM/GBSA binding energy below −60.0 kcal/mol and a toxic potential below 0.5 were considered. In order to obtain further consensus regarding candidate selection, we evaluated the binding modes of the reported compounds by visual inspection, as it is regularly performed in drug discovery projects [30,53]. For visual inspection, we chose the MD frame with the lowest binding free energy predicted by MM/GBSA calculations. This visual rating was conducted by all authors of the publication and was simply expressed by asterisks in the respective table.

## 4. Conclusions

The outbreak of SARS-CoV-2 around the globe and the resulting consequences are an impressive reminder of the menace of zoonotic diseases for public health and the economy of many countries [79]. The lack of specific therapeutics against the novel virus urges for the discovery of new drug compounds for which computational methods offer a fast and cost-efficient approach. Here, we employed a virtual screening workflow consisting of seven individual steps to ultimately determine 12 potential binders. For all reported compounds, we estimated low to moderate toxicity caused by off-target binding, as well as enhanced binding to the therapeutic target compared to the cocrystallized ligand. The reported compounds can be commercially acquired from at least one industrial vendor facilitating immediate experimental testing. In addition, we report two natural compounds, (−)-taxifolin and rhamnetin. The latter one offers a promising alternative to our other compounds and can be obtained in form of commercially available plant extracts and derived supplements. In order to provide immediate advise to ongoing clinical treatment options, we evaluated existing protease inhibitors and report an additional list of nine compounds including apixaban and nelfinavir as top hits. To this date, our study marks the most extensive computational screening for the discovery of SARS-CoV-2 main protease inhibitors. Experimental validation and subsequent optimization of our proposed early-lead compounds might offer a valuable strategy to conquer SARS-CoV-2.

## Figures and Tables

**Table 1:** Final selection of compounds compared to the cocrystallized ligand.

Compound	ΔG ^*a*^ (kcal/mol)	Score ^*b*^ (kcal/mol)	LigEff ^*c*^	logD	logS	ToxPot ^*d*^	Visual ^*e*^
CP-1	−78.2 ± 5.2	−16.7	−2.6	3.0	−4.1	0.226	***
CP-2	−75.1 ± 4.1	−15.3	−3.0	−1.9	−1.8	0.409	****
CP-3	−70.6 ± 3.9	−14.5	−2.9	0.0	−2.3	0.352	****
CP-4	−70.3 ± 5.0	−15.3	−2.6	2.5	−4.2	0.365	**
CP-5	−69.8 ± 4.2	−15.3	−2.8	3.3	−4.7	0.291	**
CP-6	−69.6 ± 3.1	−14.9	−3.7	3.1	−4.0	0.493	****
CP-7	−91.8 ± 4.1	−17.4	−2.0	11.7	−12.6	0.439	**
CP-8	−91.4 ± 5.1	−15.4	−2.5	5.9	−8.0	0.454	****
CP-9	−84.8 ± 5.1	−17.1	−2.2	8.4	−9.4	0.331	***
CP-10	−82.7 ± 4.6	−16.5	−2.2	8.3	−9.3	0.291	***
CP-11	−81.7 ± 4.8	−17.0	−2.0	5.5	−7.7	0.304	***
CP-12	−74.5 ± 4.7	−14.6	−2.1	6.0	−7.4	0.387	***
N3	−59.3 ± 7.6	−17.6	−1.6	2.3	−6.2	0.299	****
(−)-taxifolin	−53.3 ± 5.1	−16.0	−2.4	1.7	−2.1	0.289	****
rhamnetin	−52.4 ± 3.5	−16.8	−2.3	0.0	−2.1	n/a	*

^*a*^ Ligand free binding energy predicted by Molecular Mechanics/Generalized Born Surface Area (MM/GBSA) approach (excluding entropic contributions) with standard deviation; ^*b*^ Consensus docking score based on sum of highest smina and Glide scores; ^*c*^ Ligand efficiency determined from MM/GBSA score; ^*d*^ Toxic potential predicted by VirtualToxLab. ^*e*^ Quality of the binding modes from visual inspection.

**Table 2:** Final selection of repurposing compounds compared to the cocrystallized ligand and the highest scored natural compound.

Compound	ΔG (kcal/mol) ^*a*^	LigEff ^*b*^	Indication^*c*^	Approval ^*d*^	Visual ^*e*^
Apixaban	−84.0 ± 5.5	−2.5	Anticoagulant	approved	****
Nelfinavir	−80.6 ± 8.2	−2.0	Antiviral	approved	****
Glecaprevir	−80.3 ± 5.2	−1.4	Antiviral	approved	***
Lorecivivint	−79.7 ± 3.6	−1.4	Inflammation	experimental	****
Rivaroxaban	−77.2 ± 4.8	−2.7	Anticoagulant	approved	****
Betrixaban	−73.3 ± 6.0	−2.3	Anticoagulant	approved	***
Saquinavir	−71.5 ± 6.2	−1.5	Antiviral	approved	**
Voxilaprevir	−66.5 ± 5.7	−1.1	Antiviral	approved	**
Amprenavir	−66.5 ± 4.6	−1.9	Antiviral	approved	****

^*a*^ Ligand free binding energy predicted by MM/GBSA approach (excluding entropic contributions) with standard deviation; ^*b*^ Ligand efficiency determined from MM/GBSA score; ^*c*^ Pharmaceutical indication of the compound; ^*d*^ Indication and approval status derived from DrugBank [51]; ^*e*^ Quality of the binding modes from visual inspection.

## Supplementary Materials

The following are available online at https://www.mdpi.com/1422-0067/21/10/3626/s1, Figure S1: Virtual screening workflow for compounds with molecular weight above 500 g/mol, Figure S2: Structure alignment of SARS-CoV-2 crystal structures, Figure S3: Distribution of pharmacokinetically relevant descriptors for all hits supplied to the Glide SP docking protocol, Figure S4: RMSD distribution of redocking crystallographic ligands, Figure S5: Redocking of cocrystallized ligand N3, Figure S6: Binding modes of the reported compounds, Figure S7: Off-target binding profile of the reported compounds, Figure S8: Schematic depiction of the screening for drug repurposing, Table S1: Redocking of crystallographic ligands, Table S2: All hits for which MD simulations were conducted for the main screening workflow, Table S3: All hits for which MD simulations were conducted for the compounds with MW above 500 g/mol, Table S4: Number of hydrogen bonds of the proposed compounds, Table S5: Natural compounds determined in main screening, Table S6: Results from the screen for drug repurposing, Table S7: SARS-CoV inhibitors derived from the PubChem database for template selection in shape screening.
