# Supplementary material for: Potential Inhibitors for Novel Coronavirus Protease Identified by Virtual Screening of 606 Million Compounds
Source: Int J Mol Sci. 2020 May 21;21(10):3626. doi: 10.3390/ijms21103626 (PMC7279339; doi:10.3390/ijms21103626)
Supplement: Supplementary file 1 [file ijms-21-03626-s001.pdf]

Supporting Information

Potential Inhibitors for Novel Coronavirus Protease Identified by Virtual Screening of 606 Million Compounds

André Fischer, Manuel Sellner, Santhosh Neranjan, Martin Smieško*, and Markus A. Lill*,

Computational Pharmacy Group, Department of Pharmaceutical Sciences, Klingelbergstrasse 50, 4056 Basel

Corresponding author contact: martin.smiesko@unibas.ch, markus.lill@unibas.ch

Abstract: The rapid outbreak of the novel severe acute respiratory syndrome coronavirus 2 (SARS-CoV-2) in China followed by its spread around the world poses a serious global concern for public health with almost two million people tested positive and more than hundred thousand of fatalities up to date. To this date, no specific drugs or vaccines are available to treat SARS-CoV-2 despite its close relation to the SARS-CoV-1 virus that caused a similar epidemic in 2003. Thus, there remains an urgent need for the identification and development of specific antiviral therapeutics to conquer SARS-CoV-2. To conquer viral infections, the inhibition of proteases essential for proteolytic processing of viral polyproteins is a conventional therapeutic strategy. In order to find novel inhibitors, we computationally screened a compound library of over 606 million compounds for binding at the recently solved crystal structure of the main protease (M^{pro}) of SARS-CoV-2. A screening of such a vast chemical space for SARS-CoV-2 M^{pro} inhibitors has not been reported before. After shape screening, two docking protocols were applied followed by the determination of molecular descriptors relevant for pharmacokinetics to narrow down the number of initial hits. Next, molecular dynamics simulations were conducted to validate the stability of docked binding modes and comprehensively quantify ligand binding energies. After evaluation of off-target binding, we report a list of 12 purchasable compounds, with binding affinity to the target protease that is predicted to be more favorable than that of the cocrystallized peptidomimetic compound. In order to quickly advise ongoing therapeutic intervention for patients, we evaluated approved antiviral drugs and other protease inhibitors to provide a list of 9 compounds for drug repurposing. Furthermore, we identified the natural compounds (-)-taxifolin and rhamnetin as potential inhibitors of M^{pro}. Rhamnetin is already commercially available in pharmacies.

Table of Contents

Supporting Results and Discussion

Virtual Screening Procedures: Figures S1-S5, Tables S1-S3, Pharmacokinetic parameters 2

Final Selection of Compounds: Figures S6-S8, Tables S4-S6 11

Supporting Materials and Methods

Docking and shape screening: Table S7 13

Supporting References 13

Supporting Results and Discussion

Virtual screening procedures

Figure S1. Virtual screening workflow for compounds with molecular weight above 500 g/mol.

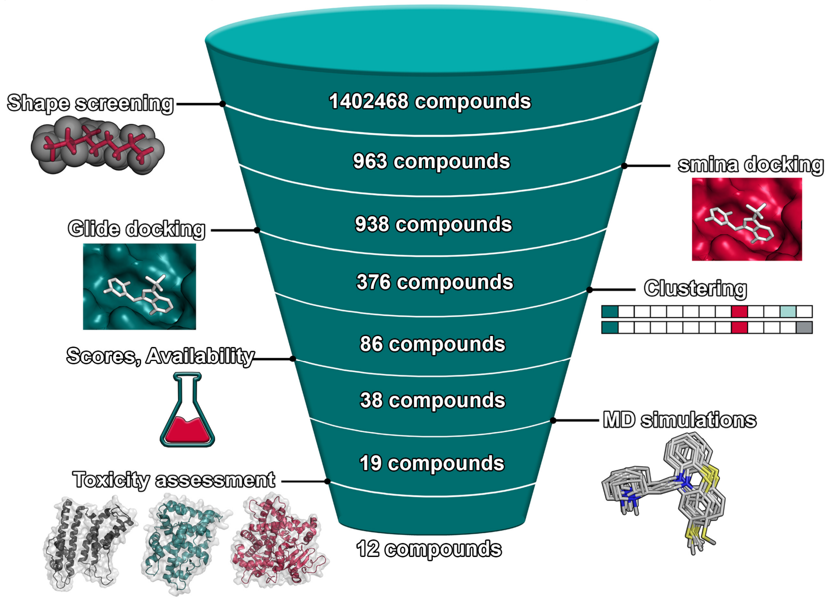

Figure S2. Structure alignment of SARS-CoV-2 crystal structures.

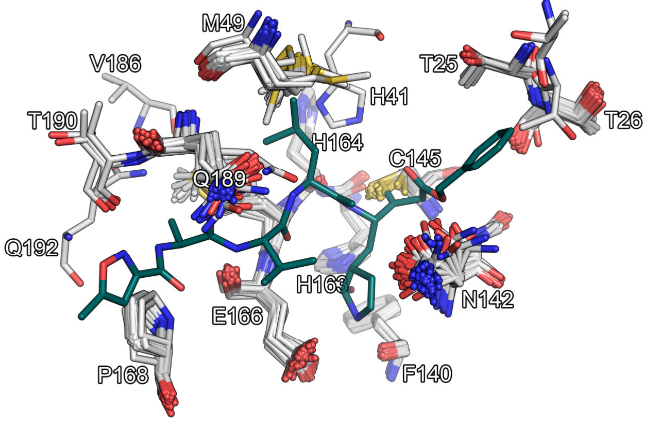

The following crystal structures were aligned: 6LU7, 6Y2G, 5M03, 5R84, 5R83, 5R82, 5R80, 5R7Z, Mpro-x0072, Mpro-x0104, Mpro-x0107, Mpro-x0161, Mpro-x0195, Mpro-x0305, Mpro-x0354, Mpro-x0387, Mpro-x0434, Mpro-x0540, Mpro-x0678, Mpro-x0689, Mpro-x0691, Mpro-x0692, Mpro-x0734, Mpro-x0749, Mpro-x0752, Mpro-x0755, Mpro-x0759, Mpro-x0769, Mpro-x0770, Mpro-x0774, Mpro-x0786, Mpro-x0820, Mpro-x0830, Mpro-x0831, Mpro-x0874, Mpro-x0946, Mpro-x0967, Mpro-x0978, Mpro-x0981, Mpro-x0991, Mpro-x0995, Mpro-x1077, Mpro-x1093, Mpro-x1249, Mpro-x1308, Mpro-x1311, Mpro-x1334, Mpro-x1336, Mpro-x1348, Mpro-x1351, Mpro-x1358, Mpro-x1374, Mpro-x1375, Mpro-x1380, Mpro-x1382, Mpro-x1384, Mpro-x1385, Mpro-x1386, Mpro-x1392, Mpro-x1402, Mpro-x1412, Mpro-x1418, Mpro-x1425, Mpro-x1458, Mpro-x1478, and Mpro-x1493. The cocrystallized ligand N3 of the protein structure 6LU7 is shown and was used to determine residues involved in ligand binding.

Figure S3. Distribution of pharmacokinetically relevant descriptors for all hits supplied to the Glide SP docking protocol.

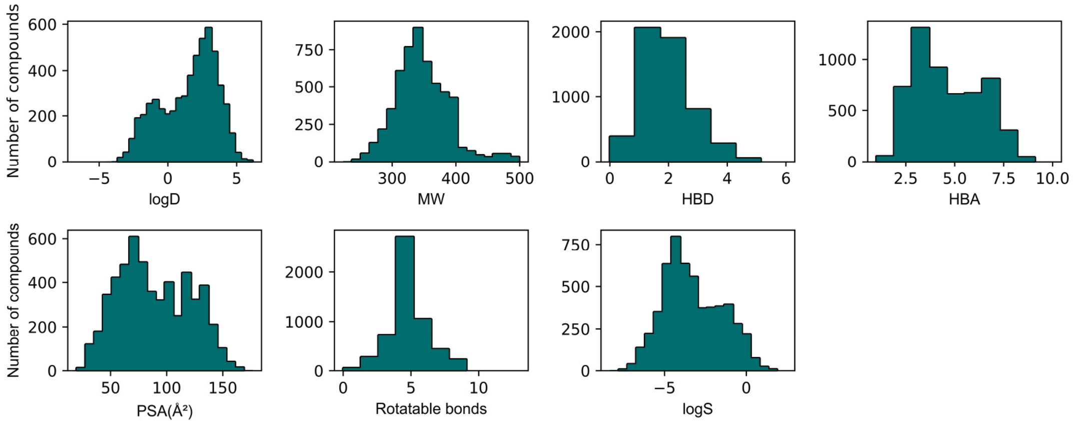

Abbreviations: HBA, hydrogen bond donors, HBD, hydrogen bond donors

Pharmacokinetic parameters

The pharmacokinetic descriptors of these compounds are shown in Figure S3. Due to our selection criterion for extracting compounds from the ZINC database, drug-likeness with respect to molecular weight (MW) was guaranteed. Several compounds, however, violated commonly accepted criteria such as the distribution coefficient logD and polar surface area (PSA). Those compounds were eliminated from further consideration. Due to the catalytic function of the target enzyme, peptides and peptidomimetics are widely applied in targeting proteases.[1] Nonetheless, disadvantages of peptides or peptidomimetics include limited oral bioavailability due to their large MW, PSA, and high number of rotatable bonds as well as poor metabolic stability and higher production cost.[2] Therefore, the development of small-molecules with balanced and favorable pharmacokinetic properties facilitating oral absorption offers a promising alternative.

Table S1. Redocking of crystallographic ligands.

Crystal structure	*Ligand 2D structure*	*RMSD Glide (Å)*	*RMSD smina (Å)*
Mpro-x0072	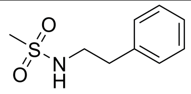	4.78	4.94
Mpro-x0104	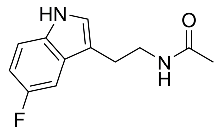	5.97	2.70
Mpro-x0107	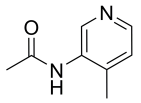	4.25	2.52
Mpro-x0161	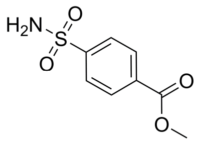	3.37	2.21
Mpro-x0195	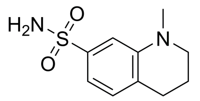	2.95	2.91
Mpro-x0305	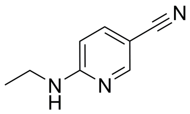	3.49	3.34
Mpro-x0354	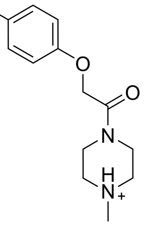	5.79	7.40
Mpro-x0387	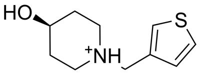	4.32	5.49
Mpro-x0434	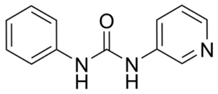	1.25	2.05
	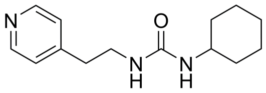	8.85	7.62
Mpro-x0678	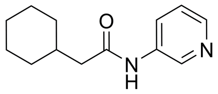	2.57	2.04

Mpro-x0689	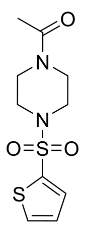	4.58	5.10
Mpro-x0691	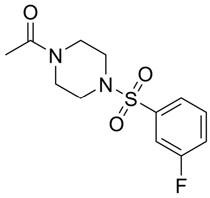	4.62	5.87
Mpro-x0692	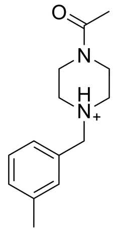	2.71	2.68
Mpro-x0734	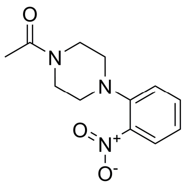	6.34	5.70
Mpro-x0749	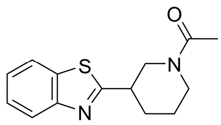	4.20	4.23
Mpro-x0752	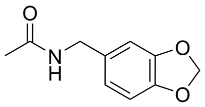	8.06	5.44
Mpro-x0755	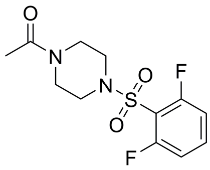	7.42	6.11
Mpro-x0759	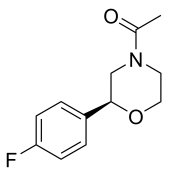	4.51	3.57
Mpro-x0769	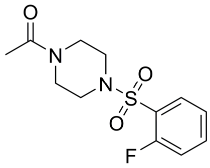	4.69	5.98
Mpro-x0770	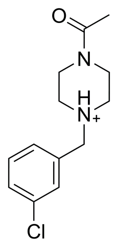	2.33	2.45

Mpro-x0774	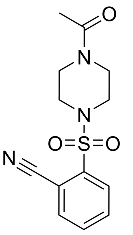	4.38	4.6
Mpro-x0786	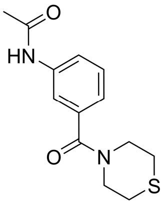	9.61	5.63
Mpro-x0820	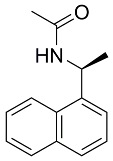	4.96	6.18
Mpro-x0830	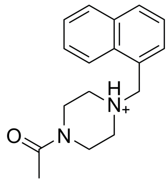	2.71	2.90
Mpro-x0831	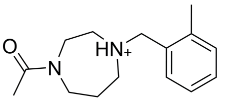	2.90	3.12
Mpro-x0874	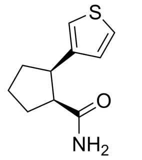	2.45	3.00
Mpro-x0946	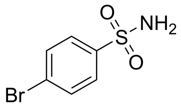	3.13	3.65
Mpro-x0967	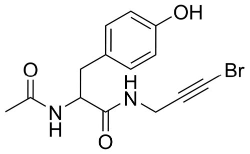	2.77	4.15
Mpro-x0978	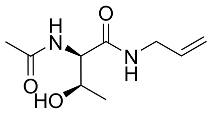	6.36	2.70
Mpro-x0981	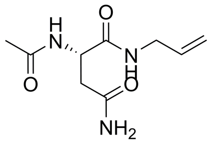	7.54	6.96
Mpro-x0991	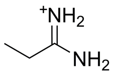	4.41	5.71
Mpro-x0995	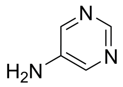	7.76	2.22
Mpro-x1077	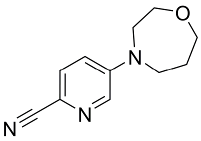	5.06	5.37

Mpro-x1093	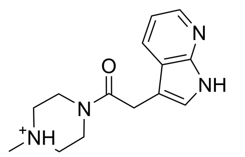	1.76	4.27
Mpro-x1249	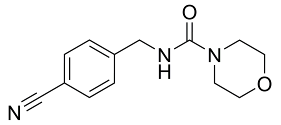	4.14	3.43
Mpro-x1308	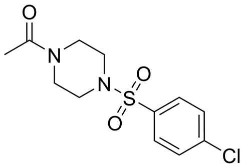	4.83	6.33
Mpro-x1311	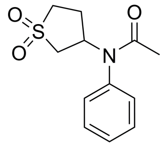	5.02	3.31
Mpro-x1334	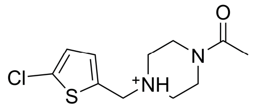	4.54	4.91
Mpro-x1336	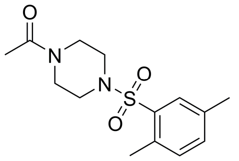	4.97	5.33
Mpro-x1348	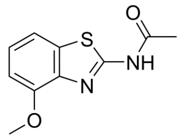	7.02	4.58
Mpro-x1351	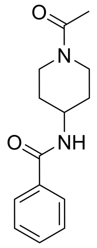	5.73	5.81
Mpro-x1358	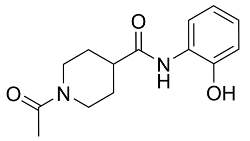	5.53	5.30
Mpro-x1374	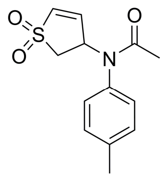	3.09	3.28
Mpro-x1375	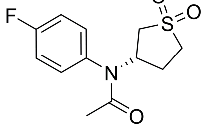	4.18	4.61
Mpro-x1380	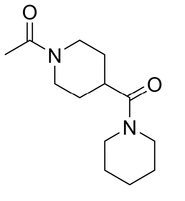	5.98	5.74

Mpro-x1382	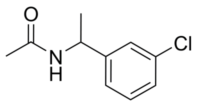	1.69	1.60
Mpro-x1384	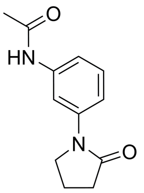	6.06	5.23
Mpro-x1385	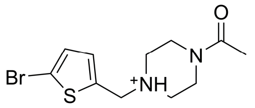	4.57	5.07
Mpro-x1386	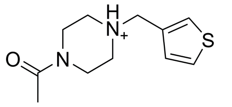	2.25	2.45
Mpro-x1392	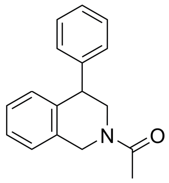	4.07	3.73
Mpro-x1402	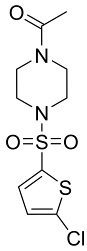	4.88	5.70
Mpro-x1412	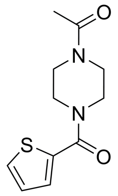	4.28	3.64
Mpro-x1418	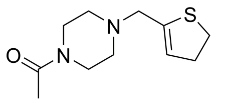	1.15	2.44
Mpro-x1425	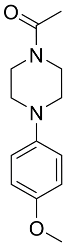	6.90	6.52
Mpro-x1458	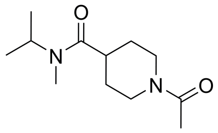	4.96	2.37
Mpro-x1478	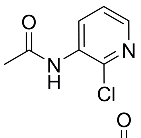	3.47	3.97
Mpro-x1493	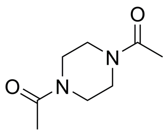	2.16	5.44

6LU7	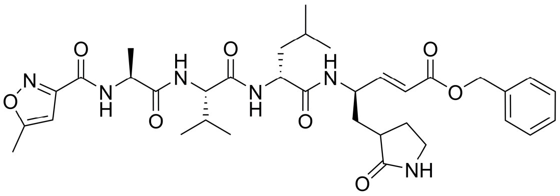	3.09	1.62
5R7Z	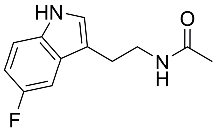	5.94	2.72
5R81	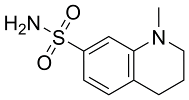	3.12	3.03
6Y2F	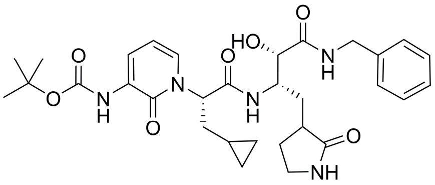	4.97	4.40
6Y7M	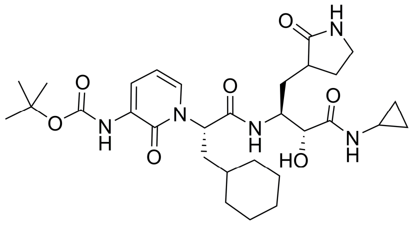	2.75	2.33

The lowest RMSD of the cocrystallized ligands compared to all five ensemble structures is reported. Crystal structures lacking the standard PDB notation were derived from the diamond webpage.[3]

Figure S4. RMSD distribution of redocking crystallographic ligands

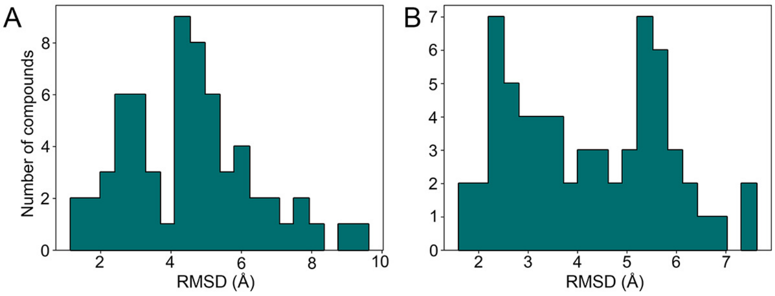

(A) RMSD distribution of the Glide SP protocol. (B) RMSD distribution of the smina protocol.

Figure S5. Redocking of cocrystallized ligand N3.

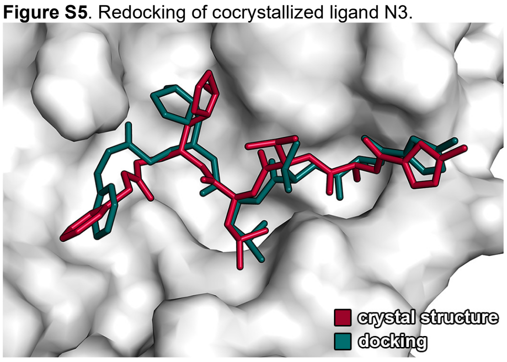

The cocrystallized ligand N3 (PDB ID 6LU7) was docked with the smina docking protocol to one of the ensemble structures.

Table S2. All hits for which MD simulations were conducted for the main screening workflow.

ZINC ID	*ΔG (kcal/mol)* [a]	*Glide score (kcal/mol)* [b]	*Smina Score (kcal/mol)* [b]	*Shape overlap*	*MW (g/mol)*	*logD*	*logS*	*PSA ($Å^2$)*	*#rot* [c]	*ToxPot* [d]	*Availability* [e]
ZINC000225850198	-80.0 ± 5.9	-7.6	-7.8	0.53	457.5	2.8	-6.0	91.7	8	0.575	In-Stock
ZINC000535149169	-78.2 ± 5.2	-8.7	-8.0	0.46	406.5	3.0	-4.1	110.3	9	0.226	On-Demand
ZINC000956196830	-75.1 ± 4.1	-7.3	-8.0	0.45	346.3	-1.9	-1.8	129.8	5	0.409	On-Demand
ZINC000409084056	-72.6 ± 5.6	-7.7	-8.3	0.52	340.4	4.7	-5.4	52.9	3	0.516	In-Stock
ZINC000078346821	-70.6 ± 3.9	-7.1	-7.4	0.53	328.4	0.0	-2.3	73.0	4	0.352	On-Demand
ZINC001564830248	-70.3 ± 5.0	-7.2	-8.0	0.55	367.4	2.5	-4.2	72.3	3	0.365	On-Demand
ZINC000436769723	-69.8 ± 4.2	-7.7	-7.6	0.51	353.4	3.3	-4.7	84.2	5	0.291	On-Demand
ZINC000067739035	-69.6 ± 3.1	-7.7	-7.3	0.58	272.7	3.1	-4.0	73.1	2	0.493	On-Demand
ZINC000582866919	-68.9 ± 4.2	-8.2	-7.7	0.54	364.4	4.2	-4.9	59.8	5	0.511	On-Demand
ZINC000751777236	-66.7 ± 4.7	-7.7	-8.2	0.53	383.4	3.8	-5.4	63.7	6	0.492	On-Demand
ZINC000437877882	-66.7 ± 4.8	-7.2	-8.3	0.46	346.4	-0.5	-2.2	122.6	6	0.102	On-Demand
ZINC000000811341	-65.3 ± 3.6	-7.4	-7.8	0.54	391.4	3.5	-6.0	105.5	6	0.494	In-Stock
ZINC000578308864	-64.6 ± 3.3	-7.2	-7.4	0.54	364.5	-0.4	-0.6	66.0	3	0.586	On-Demand
ZINC000041934593	-64.2 ± 4.9	-7.4	-7.3	0.46	336.4	1.8	-3.8	115.8	6	0.608	On-Demand
ZINC000077330923	-64.0 ± 4.5	-7.7	-7.3	0.54	320.3	2.8	-5.0	65.3	5	0.460	On-Demand
ZINC000126278505	-63.9 ± 4.1	-7.3	-7.5	0.54	336.4	2.8	-4.0	64.4	6	0.630	On-Demand
ZINC000363098575	-63.8 ± 3.6	-7.7	-7.3	0.54	324.4	3.5	-5.5	70.7	4	0.548	On-Demand
ZINC001347593526	-63.0 ± 4.7	-7.9	-7.2	0.46	331.3	-1.2	-2.8	110.5	5	0.611	For-Sale
ZINC000358067132	-62.1 ± 6.3	-8.0	-7.3	0.45	333.4	-0.9	-3.2	130.6	5	0.067	On-Demand
ZINC001189741739	-61.9 ± 6.3	-7.4	-7.8	0.45	321.3	-3.4	-1.0	137.3	4	0.195	On-Demand
ZINC000750235152	-61.8 ± 4.4	-8.0	-7.8	0.53	367.5	1.6	-4.1	71.1	7	0.198	On-Demand
ZINC000430671052	-61.7 ± 5.5	-7.4	-7.5	0.45	383.5	3.5	-5.7	62.3	4	0.308	On-Demand
ZINC000434329703	-61.7 ± 4.1	-7.2	-7.5	0.47	403.5	3.8	-4.5	68.4	6	0.318	On-Demand
ZINC001168304430	-61.3 ± 7.9	-7.6	-7.6	0.55	311.3	0.8	-2.9	73.7	5	0.471	On-Demand
ZINC000496197655	-61.3 ± 3.4	-7.4	-7.7	0.52	365.5	2.8	-3.9	36.0	4	0.475	On-Demand
ZINC001455620085	-60.9 ± 3.2	-7.2	-7.5	0.47	357.3	2.4	-4.7	88.8	6	0.538	For-Sale
ZINC001136602636	-60.9 ± 4.9	-7.8	-7.4	0.56	340.4	2.4	-3.9	64.4	6	0.604	On-Demand
ZINC000750577508	-60.4 ± 4.5	-7.4	-7.3	0.54	296.3	1.9	-4.6	70.7	4	0.588	On-Demand
ZINC000016570909	-60.3 ± 4.8	-7.6	-7.9	0.53	487.0	3.5	-5.9	73.0	5	0.291	For-Sale
ZINC001219716796	-59.8 ± 4.8	-9.0	-8.4	0.46	349.3	-2.0	-2.2	139.7	4	0.319	On-Demand
ZINC000589181351	-59.2 ± 7.3	-7.8	-7.7	0.57	376.4	3.0	-4.7	90.2	5	0.229	On-Demand
ZINC000006190212	-59.1 ± 5.2	-7.5	-7.7	0.59	374.4	3.7	-5.8	71.3	7	n/a	In-Stock
ZINC000956195718	-59.1 ± 3.9	-7.3	-7.5	0.46	319.3	-2.0	-1.4	129.9	5	n/a	On-Demand
ZINC000225779089	-59.0 ± 5.1	-7.4	-7.3	0.53	445.5	2.5	-4.7	83.8	8	n/a	In-Stock
ZINC000527718030	-58.3 ± 4.3	-7.5	-7.3	0.5	384.4	3.0	-3.8	36.8	5	n/a	On-Demand
ZINC001338948346	-58.2 ± 6.1	-7.9	-7.8	0.56	399.5	2.3	-5.2	78.8	4	n/a	On-Demand
ZINC001171153103	-58.2 ± 7.5	-7.9	-7.6	0.46	353.4	-0.2	-2.0	124.2	7	n/a	For-Sale
ZINC000096113815	-58.1 ± 4.3	-7.5	-8.3	0.56	406.4	2.4	-3.7	126.4	2	n/a	In-Stock
ZINC000128114011	-57.6 ± 4.8	-7.9	-7.2	0.54	296.4	2.5	-3.5	52.6	3	n/a	On-Demand
ZINC001610622935	-57.4 ± 4.1	-7.8	-7.7	0.54	334.4	-1.5	-0.9	96.3	5	n/a	On-Demand
ZINC000360923420	-56.8 ± 3.7	-7.6	-7.9	0.53	346.4	3.7	-5.7	59.8	4	n/a	On-Demand
ZINC000225773639	-56.8 ± 5.5	-7.3	-7.4	0.54	392.5	3.6	-5.8	67.4	7	n/a	In-Stock
ZINC000343954913	-56.7 ± 6.9	-7.4	-7.4	0.53	336.4	3.3	-4.4	32.8	4	n/a	On-Demand
ZINC001227461380	-56.6 ± 5.8	-8.3	-8.4	0.54	344.3	1.9	-2.1	116.5	3	n/a	On-Demand
ZINC000177072716	-56.3 ± 5.7	-7.9	-7.4	0.54	366.5	3.4	-5.2	70.0	5	n/a	On-Demand
ZINC000125553818	-56.1 ± 4.6	-7.1	-7.6	0.54	337.4	4.2	-5.1	64.0	4	n/a	On-Demand
ZINC000763322433	-56.0 ± 4.5	-7.6	-7.3	0.54	340.5	4.6	-5.2	70.7	5	n/a	On-Demand
ZINC000916840567	-56.0 ± 3.9	-7.2	-7.7	0.58	349.5	3.3	-4.4	35.6	4	n/a	On-Demand
ZINC001220371832	-55.9 ± 7.0	-7.4	-7.9	0.46	352.4	-2.7	-1.2	139.5	5	n/a	On-Demand
ZINC000006300080	-55.4 ± 3.9	-7.0	-7.9	0.47	411.3	2.9	-4.9	74.6	3	n/a	In-Stock
ZINC000349126162	-55.2 ± 3.7	-7.1	-7.7	0.49	338.3	2.9	-4.9	72.2	5	n/a	For-Sale
ZINC000815118295	-55.1 ± 3.5	-7.6	-7.2	0.45	318.8	2.9	-4.0	61.8	4	n/a	On-Demand
ZINC000577815676	-54.8 ± 5.0	-7.5	-7.3	0.53	304.3	2.4	-3.4	81.4	3	n/a	On-Demand
ZINC000816613055	-54.5 ± 4.3	-7.8	-7.3	0.53	289.7	2.9	-3.8	58.6	3	n/a	On-Demand
ZINC000437877883	-54.4 ± 5.6	-7.4	-7.4	0.46	346.4	-0.5	-2.2	122.6	6	n/a	On-Demand
ZINC000436920544	-54.4 ± 4.1	-7.6	-7.5	0.54	312.3	2.5	-4.7	80.2	3	n/a	On-Demand
ZINC000684931125	-54.3 ± 4.0	-7.1	-7.5	0.56	291.4	2.2	-4.9	48.1	4	n/a	For-Sale
ZINC001229703580	-54.2 ± 5.2	-8.1	-8.7	0.55	354.3	1.2	-3.1	113.3	3	n/a	On-Demand
ZINC001560407559	-54.2 ± 4.1	-8.0	-8.8	0.56	304.3	1.5	-2.0	127.5	1	n/a	In-Stock
ZINC001229703504	-54.0 ± 4.6	-8.3	-8.9	0.55	366.3	1.6	-3.8	113.3	3	n/a	On-Demand
ZINC001573475559	-54.0 ± 4.7	-7.6	-7.6	0.51	385.4	-1.3	-1.3	132.9	4	n/a	On-Demand
ZINC000000105082	-53.3 ± 5.1	-8.3	-8.5	0.55	304.3	1.7	-2.1	127.5	1	n/a	In-Stock
ZINC000479539365	-53.2 ± 5.7	-7.8	-7.2	0.55	309.4	3.4	-4.9	44.4	6	n/a	On-Demand
ZINC000624030914	-52.8 ± 4.5	-7.3	-8.3	0.46	365.4	0.1	-3.7	121.1	2	n/a	For-Sale
ZINC000336886040	-52.7 ± 3.1	-7.1	-7.4	0.56	299.8	3.4	-3.4	50.1	4	n/a	On-Demand
ZINC000502051659	-52.6 ± 5.8	-7.1	-7.7	0.49	354.4	4.1	-5.4	59.1	5	n/a	For-Sale
ZINC000596546841	-52.4 ± 3.1	-7.4	-7.6	0.53	327.7	2.5	-5.9	87.6	3	n/a	For-Sale
ZINC000016570894	-52.4 ± 4.5	-7.4	-8.0	0.53	472.9	3.0	-5.4	73.0	5	n/a	For-Sale
ZINC000003875620	-52.4 ± 3.5	-8.2	-8.6	0.67	316.3	0.0	-2.1	116.5	2	n/a	In-Stock
ZINC000614293727	-52.0 ± 6.3	-7.6	-7.3	0.56	280.3	3.7	-4.7	73.1	3	n/a	On-Demand
ZINC001168401640	-51.6 ± 3.3	-7.5	-7.4	0.53	340.3	2.1	-3.0	58.6	6	n/a	On-Demand
ZINC000059687825	-51.4 ± 4.0	-7.0	-7.7	0.59	362.4	0.1	-3.3	77.5	4	n/a	In-Stock
ZINC001610604328	-51.2 ± 5.2	-7.6	-7.5	0.55	312.4	-2.0	0.0	77.4	5	n/a	On-Demand
ZINC000290051168	-51.0 ± 3.7	-7.7	-7.7	0.54	279.3	3.7	-5.0	38.3	3	n/a	On-Demand
ZINC001045216116	-50.9 ± 6.0	-7.7	-7.5	0.53	348.4	0.2	-2.7	81.6	5	n/a	On-Demand
ZINC000224103212	-50.8 ± 3.6	-7.2	-7.5	0.52	475.6	3.5	-5.0	65.9	7	n/a	In-Stock
ZINC000779067247	-50.8 ± 5.3	-7.4	-7.2	0.52	298.3	2.8	-3.2	58.3	5	n/a	On-Demand
ZINC000065385626	-50.7 ± 3.2	-7.4	-7.3	0.6	310.3	-1.3	-1.0	85.4	5	n/a	In-Stock
ZINC001450073782	-50.6 ± 2.7	-7.2	-7.6	0.46	296.3	1.1	-3.8	126.7	4	n/a	For-Sale
ZINC000065385629	-50.6 ± 4.2	-7.2	-7.4	0.6	310.3	-1.3	-1.0	85.4	5	n/a	In-Stock
ZINC000833074436	-50.5 ± 3.9	-7.4	-7.5	0.57	271.3	-0.8	-0.3	93.0	3	n/a	On-Demand
ZINC001219365937	-50.3 ± 8.0	-7.5	-8.1	0.45	338.4	-2.7	-1.1	139.5	4	n/a	On-Demand
ZINC000154873179	-50.2 ± 3.8	-7.6	-7.8	0.46	351.4	0.3	-3.1	127.8	7	n/a	On-Demand
ZINC000035426457	-50.1 ± 4.7	-7.3	-7.3	0.57	271.3	-0.8	-0.4	93.0	3	n/a	On-Demand
ZINC000117113459	-50.0 ± 4.5	-7.5	-7.3	0.54	328.4	3.9	-5.9	71.5	5	n/a	On-Demand
ZINC000940345878	-49.7 ± 4.1	-8.1	-7.7	0.47	345.4	-2.0	-2.0	119.7	4	n/a	On-Demand
ZINC000940345761	-49.6 ± 4.1	-7.8	-7.4	0.45	331.4	-2.1	-2.4	119.7	4	n/a	On-Demand

ZINC001324291630	-49.5 ± 4.4	-7.1	-7.5	0.47	335.4	-0.9	-2.4	139.2	5	n/a	On-Demand
ZINC000128113531	-49.4 ± 4.9	-7.4	-7.5	0.54	331.4	2.9	-4.4	45.2	3	n/a	On-Demand
ZINC000667524349	-49.3 ± 3.8	-7.2	-7.4	0.54	342.4	-0.5	-1.4	103.7	4	n/a	On-Demand
ZINC000347640761	-49.3 ± 3.4	-7.3	-7.5	0.53	295.3	2.4	-3.5	72.0	3	n/a	For-Sale
ZINC001627243052	-49.3 ± 3.9	-7.2	-7.7	0.54	349.4	2.9	-4.7	65.4	4	n/a	On-Demand
ZINC000437857922	-47.8 ± 8.6	-7.3	-7.5	0.45	344.4	1.0	-4.0	113.3	6	n/a	On-Demand
ZINC001209530875	-47.8 ± 5.2	-7.3	-7.3	0.48	351.4	-1.9	-1.1	130.6	8	n/a	On-Demand
ZINC001009575024	-47.8 ± 4.2	-7.9	-7.4	0.55	370.5	2.6	-4.8	58.6	4	n/a	On-Demand
ZINC001218424555	-47.4 ± 4.3	-8.1	-8.4	0.54	370.4	1.3	-2.5	116.5	4	n/a	On-Demand
ZINC000068759657	-47.3 ± 5.8	-7.2	-7.6	0.53	382.9	3.3	-5.3	76.0	5	n/a	On-Demand
ZINC000543440359	-47.3 ± 3.1	-7.0	-7.5	0.54	334.8	4.1	-5.8	59.1	3	n/a	For-Sale
ZINC000778587708	-47.2 ± 5.6	-7.6	-7.4	0.59	323.4	2.1	-4.0	75.4	5	n/a	On-Demand
ZINC001170115101	-46.9 ± 5.7	-7.4	-7.5	0.53	338.3	-0.4	-0.6	88.1	5	n/a	On-Demand
ZINC000488902540	-46.3 ± 5.0	-7.4	-7.8	0.52	301.3	3.1	-5.4	74.9	3	n/a	For-Sale
ZINC000733546084	-46.2 ± 4.5	-7.4	-8.1	0.47	375.4	-0.1	-2.7	131.4	6	n/a	On-Demand
ZINC001189645413	-45.8 ± 4.3	-7.4	-7.5	0.53	312.2	1.5	-2.6	69.6	3	n/a	On-Demand
ZINC000466425235	-45.7 ± 4.5	-7.7	-7.6	0.57	338.3	3.2	-5.0	32.3	5	n/a	For-Sale
ZINC001368308700	-45.5 ± 3.7	-7.4	-7.5	0.52	334.4	0.8	-1.1	96.2	5	n/a	For-Sale
ZINC000732955887	-45.3 ± 5.1	-7.9	-7.6	0.54	349.3	0.9	-4.9	100.4	5	n/a	On-Demand
ZINC000732955886	-45.3 ± 5.1	-7.9	-7.6	0.54	349.3	0.9	-4.9	100.4	5	n/a	On-Demand
ZINC000001689531	-44.8 ± 4.4	-8.2	-8.5	0.55	304.3	1.0	-2.1	127.5	1	n/a	In-Stock
ZINC000347488294	-44.7 ± 2.4	-7.3	-7.5	0.53	294.3	3.2	-4.8	59.1	3	n/a	For-Sale
ZINC000005733537	-44.7 ± 4.6	-7.8	-7.5	0.53	314.3	0.6	-3.1	85.2	3	n/a	In-Stock
ZINC001410147625	-44.5 ± 3.2	-7.4	-7.3	0.47	325.3	-0.4	-2.2	133.1	7	n/a	For-Sale
ZINC001123890204	-44.0 ± 6.2	-7.9	-7.7	0.48	356.4	-1.9	0.0	91.3	6	n/a	On-Demand
ZINC000553090364	-43.9 ± 4.2	-7.2	-7.3	0.49	363.4	2.2	-4.1	102.9	7	n/a	On-Demand
ZINC001088166116	-43.7 ± 8.1	-7.3	-7.9	0.47	354.5	-1.2	-0.3	66.3	6	n/a	On-Demand
ZINC000003775158	-43.6 ± 4.6	-8.6	-8.5	0.6	286.2	0.6	-2.6	107.2	1	n/a	In-Stock
ZINC000512344696	-43.5 ± 4.2	-7.4	-8.0	0.54	393.6	2.5	-3.2	40.0	6	n/a	On-Demand
ZINC000983531992	-43.0 ± 2.8	-7.5	-7.6	0.46	345.4	-1.7	-1.7	128.5	5	n/a	On-Demand
ZINC001225625327	-42.5 ± 5.2	-8.1	-8.6	0.54	379.4	2.1	-2.8	120.0	3	n/a	On-Demand
ZINC001217945397	-42.0 ± 4.8	-8.2	-8.2	0.53	400.4	1.4	-3.5	81.6	4	n/a	On-Demand
ZINC000524731490	-41.8 ± 2.7	-7.2	-8.7	0.54	443.4	-2.0	-1.5	136.4	4	n/a	In-Stock
ZINC001262800712	-41.6 ± 6.1	-7.4	-7.4	0.57	399.5	2.3	-5.2	78.8	4	n/a	On-Demand
ZINC001227462040	-41.4 ± 4.6	-8.2	-8.5	0.56	394.4	2.5	-3.3	116.5	4	n/a	On-Demand
ZINC000348315879	-41.2 ± 5.1	-7.2	-7.3	0.54	312.3	2.7	-3.7	58.3	6	n/a	On-Demand
ZINC001026583781	-41.2 ± 8.0	-7.7	-7.9	0.46	356.4	-0.8	-3.2	119.9	4	n/a	On-Demand
ZINC000407986242	-41.1 ± 3.7	-8.2	-8.0	0.55	277.3	2.8	-4.9	62.3	1	n/a	In-Stock
ZINC000095540481	-41.0 ± 4.0	-7.1	-7.7	0.54	326.4	3.0	-4.8	80.2	3	n/a	On-Demand
ZINC000547274434	-41.0 ± 4.6	-7.2	-7.4	0.55	411.3	-0.4	-2.1	74.1	4	n/a	On-Demand
ZINC001227460663	-40.8 ± 6.1	-8.3	-8.7	0.56	384.4	2.8	-3.1	116.5	3	n/a	On-Demand
ZINC000096114144	-40.7 ± 3.8	-7.4	-8.5	0.53	420.4	2.6	-3.8	126.4	2	n/a	In-Stock
ZINC000061624333	-39.5 ± 5.0	-7.4	-7.8	0.53	399.5	3.8	-5.6	75.4	6	n/a	On-Demand
ZINC000614293483	-39.4 ± 3.8	-7.6	-7.5	0.58	278.3	3.4	-4.6	73.1	2	n/a	On-Demand
ZINC000532472539	-39.4 ± 4.4	-7.1	-7.6	0.48	358.5	2.3	-4.8	75.1	6	n/a	On-Demand
ZINC000152703551	-38.7 ± 3.8	-7.0	-7.5	0.5	360.2	3.2	-2.9	67.6	5	n/a	For-Sale
ZINC000095416842	-38.1 ± 3.9	-7.6	-7.3	0.46	349.4	1.8	-3.7	102.9	7	n/a	For-Sale
ZINC000014726551	-37.6 ± 4.6	-7.0	-8.8	0.57	300.2	2.4	-3.5	120.4	0	n/a	In-Stock
ZINC000079763441	-37.3 ± 6.2	-7.4	-7.4	0.5	364.4	2.6	-4.2	115.8	7	n/a	On-Demand
ZINC001285432327	-35.7 ± 6.1	-7.8	-7.3	0.53	348.3	-2.1	-1.2	138.2	8	n/a	For-Sale
ZINC001220589794	-34.2 ± 6.1	-7.1	-7.4	0.47	370.4	-1.0	-2.0	136.6	5	n/a	On-Demand
ZINC001324752707	-32.9 ± 5.3	-8.5	-7.3	0.46	305.3	-1.0	-2.5	110.5	5	n/a	On-Demand
ZINC001193635195	-30.7 ± 5.5	-7.3	-7.2	0.58	255.2	0.1	-0.8	106.2	2	n/a	On-Demand
ZINC000646465837	-29.4 ± 4.9	-7.4	-7.4	0.51	339.4	0.9	-3.6	88.1	4	n/a	On-Demand
ZINC001141388515	-27.7 ± 5.6	-7.5	-7.2	0.56	329.7	0.8	-4.2	90.5	3	n/a	On-Demand
ZINC001013273066	-26.5 ± 4.7	-7.4	-7.5	0.45	328.3	-0.8	-2.7	131.2	3	n/a	On-Demand
ZINC001175098626	-16.0 ± 4.3	-7.5	-7.4	0.54	386.5	2.7	-4.7	95.8	5	n/a	For-Sale

Compounds in italics are hits reported in the main text. For some compounds no toxicity assessment was performed (n/a).
[a]Ligand free binding energy predicted by MM/GBSA approach (excluding entropic contributions) with standard deviation;
[b]Lowest docking score from docking against ensemble of five structures;
[c]Number of rotatable bonds;
[d]Toxic potential determined in the VirtualToxLab;
[e]Availability of the compounds according to the ZINC database.[4]

Table S3. All hits for which MD simulations were conducted for the compounds with MW above 500 g/mol.

ZINC ID	*ΔG (kcal/mol)* [a]	*Glide score (kcal/mol)* [b]	*Smina Score (kcal/mol)* [b]	*Shape overlap*	*MW (g/mol)*	*logD*	*logS*	*PSA (Å^2)*	*#rot* [c]	*ToxPot* [d]	*Availability* [e]
ZINC000072130459	-95.2 ± 4.1	-7.0	-9.0	0.65	556.6	7.4	-8.8	74.2	10	0.685	In-Stock
ZINC000020731346	-93.6 ± 4.7	-7.6	-7.9	0.46	508.4	4.8	-8.0	53.1	4	0.553	For-Sale
ZINC000098047142	-91.8 ± 4.1	-8.2	-9.1	0.47	747.3	11.7	-12.6	54.0	10	0.439	In-Stock
ZINC000225252997	-91.4 ± 5.1	-7.7	-7.7	0.46	500.6	5.9	-8.0	67.4	9	0.454	In-Stock
ZINC000103644480	-88.3 ± 4.0	-7.4	-8.6	0.46	550.6	8.5	-10.1	65.3	5	0.536	For-Sale
ZINC000409081785	-84.8 ± 5.1	-8.6	-8.5	0.52	672.8	8.4	-9.4	76.0	6	0.331	In-Stock
ZINC000097944956	-84.5 ± 5.0	-7.4	-8.6	0.46	550.6	8.5	-10.1	65.3	5	0.536	In-Stock
ZINC000002357423	-83.6 ± 5.2	-7.2	-8.8	0.46	513.9	6.6	-9.0	63.2	5	0.623	In-Stock
ZINC000409079528	-82.7 ± 4.6	-8.5	-8.1	0.50	652.4	8.3	-9.3	76.0	6	0.291	In-Stock
ZINC000036158243	-82.5 ± 5.9	-7.4	-7.9	0.49	520.4	2.6	-5.8	87.7	8	0.477	For-Sale
ZINC000100708038	-81.7 ± 4.8	-8.2	-8.8	0.48	553.6	5.5	-7.7	106.5	7	0.304	In-Stock
ZINC000034736583	-79.6 ± 3.8	-7.7	-8.1	0.45	502.8	6.2	-8.5	52.7	5	0.525	For-Sale
ZINC000004195875	-78.3 ± 4.9	-7.5	-8.5	0.46	516.4	7.0	-8.2	54.8	5	0.507	In-Stock
ZINC000035689851	-74.5 ± 4.7	-7.2	-7.5	0.46	508.4	6.0	-7.4	70.1	8	0.387	For-Sale
ZINC000013575045	-73.0 ± 4.2	-7.8	-7.7	0.49	513.7	3.7	-8.0	83.8	8	0.367	In-Stock
ZINC000102922714	-72.6 ± 7.3	-7.6	-8.4	0.47	618.5	6.6	-9.2	83.1	9	0.414	In-Stock
ZINC000011757999	-72.3 ± 5.7	-8.2	-8.1	0.45	539.6	6.0	-7.3	97.3	9	0.204	In-Stock
ZINC000098021319	-72.0 ± 4.9	-7.5	-7.2	0.46	552.9	6.2	-7.6	70.1	8	0.374	For-Sale
ZINC000097946172	-70.0 ± 7.4	-7.5	-8.9	0.48	572.7	8.7	-9.2	59.7	8	0.378	In-Stock
ZINC000100415333	-69.6 ± 4.5	-7.0	-8.9	0.45	570.3	5.1	-7.8	80.8	3	n/a	In-Stock
ZINC000000788070	-68.2 ± 6.0	-7.1	-8.2	0.45	500.3	5.6	-6.2	67.4	8	n/a	In-Stock
ZINC000102973979	-67.3 ± 5.1	-7.4	-8.6	0.56	584.3	6.9	-9.1	63.0	4	n/a	In-Stock

ZINC000097971922	-66.7 ± 6.5	-8.2	-7.9	0.48	584.6	3.0	-4.1	163.0	10	n/a	In-Stock
ZINC000150383182	-64.7 ± 5.7	-7.1	-8.4	0.46	667.8	2.9	-4.5	133.8	16	n/a	In-Stock
ZINC000223764140	-64.7 ± 6.2	-7.5	-7.9	0.47	519.1	7.4	-9.3	58.2	8	n/a	In-Stock
ZINC000036158329	-64.0 ± 6.1	-7.5	-8.3	0.50	504.4	3.4	-6.5	78.5	6	n/a	For-Sale
ZINC000103418421	-60.4 ± 5.1	-8.3	-9.1	0.46	550.6	6.1	-7.4	55.8	5	n/a	For-Sale
ZINC000012412803	-60.0 ± 5.5	-7.3	-8.1	0.47	504.4	3.0	-2.2	74.9	5	n/a	In-Stock
ZINC000003408051	-58.2 ± 4.6	-7.4	-7.2	0.47	533.2	5.5	-7.8	104.0	6	n/a	In-Stock
ZINC000103120251	-58.1 ± 5.6	-7.8	-8.1	0.48	534.4	5.3	-7.0	75.5	6	n/a	For-Sale
ZINC000100004777	-57.4 ± 4.6	-7.7	-8.5	0.49	525.4	7.3	-9.0	61.4	4	n/a	In-Stock
ZINC000102757914	-55.7 ± 4.6	-7.8	-8.3	0.50	539.8	6.7	-9.0	63.0	4	n/a	In-Stock
ZINC000103140857	-55.7 ± 5.3	-7.8	-7.9	0.48	538.4	5.2	-7.3	75.5	6	n/a	For-Sale
ZINC000408583162	-53.2 ± 6.1	-7.3	-7.8	0.45	532.2	7.1	-9.0	42.9	6	n/a	In-Stock
ZINC000100408112	-51.0 ± 7.0	-7.1	-8.5	0.48	569.4	8.6	-9.5	72.5	6	n/a	In-Stock
ZINC000034735622	-47.5 ± 3.1	-7.5	-7.7	0.45	501.9	5.9	-8.1	52.7	6	n/a	For-Sale
ZINC000101601966	-44.0 ± 3.6	-9.2	-9.3	0.45	569.6	-1.0	-0.6	142.2	7	n/a	In-Stock
ZINC000514383436	-42.6 ± 3.9	-7.5	-8.0	0.46	572.6	4.5	-7.1	110.5	10	n/a	On-Demand

Compounds in italics are hits reported in the main text. For some compounds no toxicity assessment was performed (n/a).
[a]Ligand free binding energy predicted by MM/GBSA approach (excluding entropic contributions) with standard deviation;
[b]Lowest docking score from docking against ensemble of five structures;
[c]Number of rotatable bonds;
[d]Toxic potential determined in the VirtualToxLab;
[e]Availability of the compounds according to the ZINC database.[4]

Final Selection of Compounds

Figure S6. Binding modes of the reported compounds.

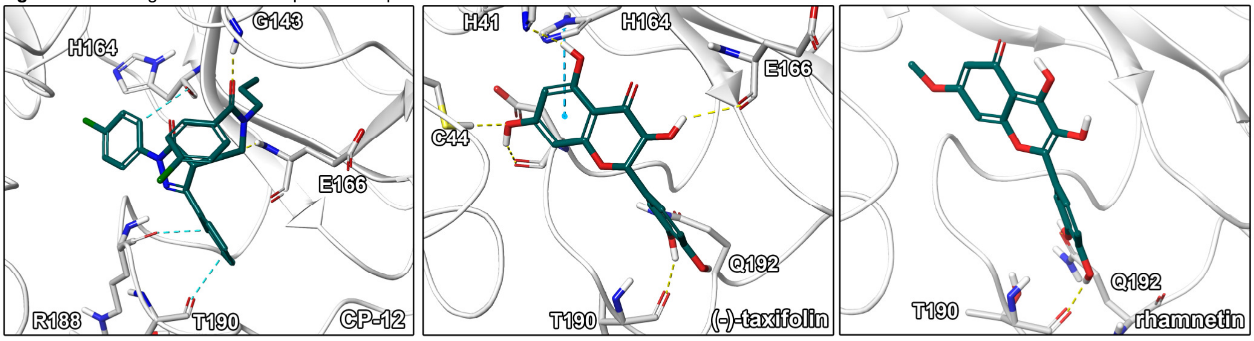

The binding modes were assessed for the MD frame with lowest binding free energy according to MM/GBSA post-processing.

Table S4. Number of hydrogen bonds of the proposed compounds.

Compound	*Hydrogen bonds*
Apixaban	3
Nelfinavir	7
Glecaprevir	4
Lorecivivint	5
Rivaroxaban	5
Betrixaban	1
Saquinavir	4
Voxilaprevir	3
Amprenavir	4
Average repurposing	4.0
CP-1	5
CP-2	5
CP-3	3
CP-4	1
CP-5	3
CP-6	5
CP-7	2
CP-8	4
CP-9	3
CP-10	3
CP-11	2
CP-12	2
(-)-taxifolin	6
Average shape screen	3.4

The number of hydrogen bonds was determined for the MD frame with lowest binding free energy.

Table S5. Natural compounds determined in main screening.

ZINC ID	*Trivial name*	*ΔG (kcal/mol)* [a]
ZINC000000105082	(-)-taxifolin	-53.3 ± 5.1
ZINC000003875620	Rhamnetin	-52.4 ± 3.5
n/a	(+)-taxifolin	-39.5 ± 5.7

[a]Ligand free binding energy predicted by MM/GBSA approach (excluding entropic contributions) with standard deviation.

Figure S7. Off-target binding profile of the reported compounds.

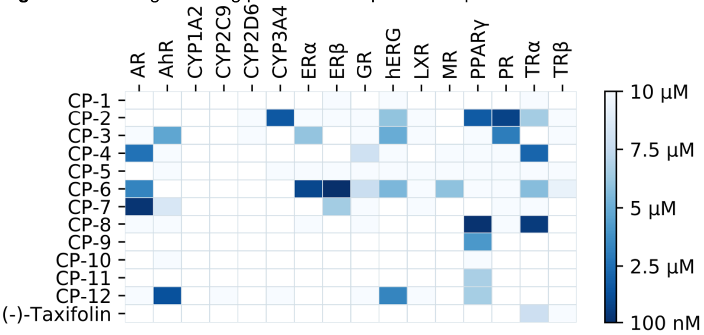

The off-target binding profile was obtained from the VirtualToxLab.[5,6]

Figure S8. Schematic depiction of the screening for drug repurposing.

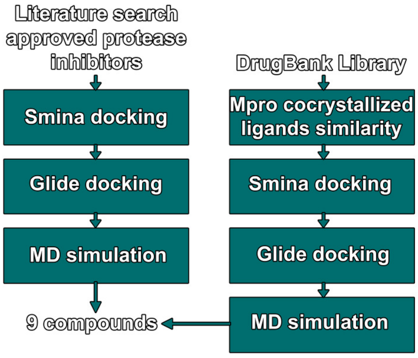

Table S6. Results from the screen for drug repurposing.

Trivial Name	*DrugBank ID*	*Indication* [a]	*ΔG (kcal/mol)* [b]	*Glide score (kcal/mol)* [c]	*Smina Score (kcal/mol)* [c]	*Origin* [d]	*Approval status* [e]
Apixaban	DB06605	Anticoagulant	-84.0 ± 5.5	-7.7	-8.7	Literature	yes
Nelfinavir	DB00220	Antiviral	-80.6 ± 8.2	-8.4	-9.2	Literature	yes
Glecaprevir	DB13879	Antiviral	-80.3 ± 5.2	-5.9	-9.1	Literature	yes
Lorecivivint	DB14883	Inflammation	-79.7 ± 5.0	-7.0	-9.2	Similarity search	no
Rivaroxaban	DB06228	Anticoagulant	-77.2 ± 4.1	-6.8	-7.9	Literature	yes
Betrixaban	DB12364	Anticoagulant	-73.3 ± 3.8	-7.0	-8.1	Literature	yes
Saquinavir	DB01232	Antiviral	-71.5 ± 8.8	-8.2	-8.7	Literature	yes
Voxilaprevir	DB12026	Antiviral	-66.5 ± 4.6	-7.2	-8.3	Literature	yes
Amprenavir	DB00701	Antiviral	-66.5 ± 6.8	-6.7	-7.4	Literature	yes
Telaprevir	DB05521	Antiviral	-63.3 ± 7.7	-7.6	-7.5	Literature	yes
Simeprevir	DB06290	Antiviral	-62.4 ± 3.7	-8.1	-9.0	Literature	yes
Darunavir	DB01264	Antiviral	-61.4 ± 6.9	-7.5	-8.2	Similarity search	yes
n/a	DB07991	Antiestrogen	-61.4 ± 5.1	-7.5	-7.8	Similarity search	no
(R)-boceprevir	DB08873	Antiviral	-60.7 ± 5.8	-7.2	-7.6	Literature	yes
N3	n/a	Antiviral	-59.3 ± 7.6	-9.9	-7.7	Protein Data Bank	no
n/a	DB01810	Cancer	-58.8 ± 4.4	-8.6	-7.3	Similarity search	no
n/a	DB06890	Antifungal	-55.7 ± 3.2	-6.2	-5.9	Similarity search	no
Asunaprevir	DB11586	Antiviral	-55.1 ± 5.8	-6.3	-8.0	Literature	yes
n/a	DB07222	Unknown	-53.6 ± 4.0	-6.6	-6.8	Similarity search	no
Tipranavir	DB00932	Antiviral	-52.8 ± 4.4	-6.0	-8.3	Literature	yes
Indinavir	DB00224	Antiviral	-52.4 ± 6.0	-6.5	-8.5	Literature	yes
Difenpiramide	DB13371	Unknown	-52.0 ± 4.5	-6.2	-7.1	Similarity search	no
Grazoprevir	DB11575	Antiviral	-51.1 ± 7.1	-6.1	-8.9	Literature	yes
Topiroxostat	DB01685	Unknown	-50.5 ± 3.6	-6.4	-7.1	Similarity search	no
(S)-boceprevir	DB08873	Antiviral	-49.7 ± 3.9	-7.4	-7.8	Literature	yes
n/a	DB07781	Unknown	-48.0 ± 3.6	-5.4	-6.4	Similarity search	no
Atazanavir	DB01072	Antiviral	-47.8 ± 9.2	-6.4	-7.3	Literature	yes
Pyroxamide	DB12847	Unknown	-47.3 ± 3.9	-3.0	-6.3	Similarity search	no
MSX-122	DB12715	Unknown	-46.7 ± 4.4	-4.8	-6.5	Similarity search	no
Paritaprevir	DB09297	Antiviral	-46.4 ± 6.0	-8.1	-9.5	Literature	yes
Lopinavir	DB01601	Antiviral	-46.1 ± 8.3	-8.9	-7.8	Literature	yes
Ritonavir	DB00503	Antiviral	-45.8 ± 7.5	-7.5	-8.0	Literature	yes
n/a	DB07152	Unknown	-44.4 ± 4.1	-6.4	-8.8	Similarity search	no
N-acetylserotonin	DB04275	Unknown	-41.3 ± 5.2	-6.6	-6.3	Similarity search	no
n/a	DB04601	Unknown	-38.2 ± 4.1	-6.8	-7.4	Similarity search	no
Dapivirine	DB08639	Antiviral	-36.6 ± 3.0	-6.5	-7.4	Similarity search	no
Dansylamide	DB02866	Unknown	-35.5 ± 4.1	-5.5	-5.9	Similarity search	no
Melatonin	DB01065	Insomnia	-33.7 ± 3.3	-5.9	-5.9	Similarity search	yes
Indane-5-sulfonamide	DB08165	Unknown	-28.2 ± 2.8	-6.3	-5.7	Similarity search	no
Fosamprenavir	DB01319	Antiviral	-25.3 ± 4.3	-6.9	-7.6	Literature	yes
n/a	DB03468	Unknown	-21.9 ± 3.5	-6.2	-5.9	Similarity search	no
n/a	DB07114	Unknown	-14.6 ± 4.8	-5.8	-5.6	Similarity search	no

[a]Indication retrieved from the DrugBank database;[7]
[b]Ligand free binding energy predicted by MM/GBSA approach (excluding entropic contributions) with standard deviation;
[c]Lowest docking score from docking against ensemble of five structures;
[d]Workflow from which the compound was selected for MD;
[e]Approval status of the compound according to the DrugBank database.[7]

Supporting Materials and Methods

Docking and shape screening

Table S7. SARS-CoV-1 inhibitors derived from the PubChem database for template selection in shape screening.

PubChem Compound ID	*Activity type*	*Activity (μM)*	*Method*	*PubChem bioassay ID*	*Selection[a]*
127045229	IC_{50}	6.7	Fluorimetric assay	1304461	no
127045221	IC_{50}	8.6	Fluorimetric assay	1304461	no
127043644	IC_{50}	6.4	Fluorimetric assay	1304461	no
127043642	IC_{50}	5.8	Fluorimetric assay	1304461	yes
127043641	IC_{50}	6.0	Fluorimetric assay	1304461	no
25256829	IC_{50}	5.4	FRET	417985	yes
45271832	IC_{50}	8.1	FRET	430140	no
45271831	IC_{50}	9.3	FRET	430140	no
45271826	IC_{50}	5.2	FRET	430140	no
45270979	IC_{50}	4.1	FRET	430140	yes
44517748	IC_{50}	2.7	FRET	430140	no
5280343	IC_{50}	8.1	FRET	430140	no
2131982	K_i	9.9	FRET	596993	no
2131972	K_i	9.1	FRET	596993	yes

[a]Depiction if compound was used in shape screening.

Supporting References

(1) Pillaiyar, T.; Manickam, M.; Namasivayam, V.; Hayashi, Y.; Jung, S. H. An Overview of Severe Acute Respiratory Syndrome-Coronavirus (SARS-CoV) 3CL Protease Inhibitors: Peptidomimetics and Small Molecule Chemotherapy. *J. Med. Chem.* **2016**, *59*, 6595–6628.

(2) Recio, C.; Maione, F.; Iqbal, A. J.; Mascolo, N.; De Feo, V. The Potential Therapeutic Application of Peptides and Peptidomimetics in Cardiovascular Disease. *Front. Pharmacol.* **2017**, *7*, 1–11.

(3) Diamond. Main protease structure and XChem fragment screen https://www.diamond.ac.uk/covid-19/for-scientists/Main-protease-structure-and-XChem/Downloads.html (accessed Mar 20, 2020).

(4) Sterling, T.; Irwin, J. J. ZINC 15 - Ligand Discovery for Everyone. *J. Chem. Inf. Model.* **2015**, *55*, 2324–2337.

(5) Vedani, A.; Dobler, M.; Smieško, M. VirtualToxLab - A Platform for Estimating the Toxic Potential of Drugs, Chemicals and Natural Products. *Toxicol. Appl. Pharmacol.* **2012**, *261*, 142–153.

(6) Vedani, A.; Dobler, M.; Hu, Z.; Smieško, M. OpenVirtualToxLab-A Platform for Generating and Exchanging in Silico Toxicity Data. *Toxicol. Lett.* **2015**, *232*, 519–532.

(7) Wishart, D. S.; Feunang, Y. D.; Guo, A. C.; Lo, E. J.; Marcu, A.; Grant, J. R.; Sajed, T.; Johnson, D.; Li, C.; Sayeeda, Z.; et al. DrugBank 5.0: A Major Update to the DrugBank Database for 2018. *Nucleic Acids Res.* **2018**, *46*, D1074–D1082.
